# Immune remodeling and myeloid signature in the bone marrow niche of *MYCN*-driven neuroblastoma

**DOI:** 10.1038/s44276-026-00249-3

**Published:** 2026-09-15

**Authors:** Bethel Tesfai Embaie, Adele M. Alchahin, Lilly Velentza, Thale Kristin Olsen, Ioana Maria Gavriliuc, Shenglin Mei, Ninib Baryawno

**Affiliations:** 1 Division of Pediatric Oncology and Pediatric Surgery, Department of Women’s and Children’s Health, Karolinska Institutet, SE 171 77 Stockholm, Sweden; 2 Department of Immunology, Genetics and Pathology, Uppsala University, SE 751 85 Uppsala, Sweden; 3 Fralin Biomedical Research Institute, Virginia Tech FBRI Cancer Research Center, Washington, DC USA; 4 Department of Biomedical Sciences and Pathobiology, College of Veterinary Medicine, Virginia Tech, Blacksburg, VA USA

## Abstract

**Background:**

High-risk *MYCN*-amplified neuroblastoma is associated with a dire prognosis and often metastasizes to the bone marrow. However, systemic immune remodeling and its effect on the bone marrow niche remain unclear.

**Methods:**

Harnessing single-cell RNA sequencing, we compare the bone marrow immune cell landscape of wild-type (WT) mice and TH-*MYCN* transgenic mice.

**Results:**

TH-*MYCN* mice showed increased neutrophils and *Cd300e*+ myeloid cells, and reduced developing B cells, mirroring our findings in human neuroblastoma bone marrow metastases. Ligand-receptor analysis revealed enrichment of ADGRG and SEMA4 signaling pathways in TH-*MYCN* bone marrow, highlighting Tgm2-Adgrg1, Sema4D-Plxnb2, and Sema4D-Cd72 interactions as potential immune modulators. In contrast, CD45 and CEACAM pathways were prominent in WT mice, suggesting context-specific immune regulation. A distinct *Cd300e*+ *Pecam1*+ myeloid population enriched in TH-*MYCN* mice, exhibited a gene signature, including *Fabp4*, *Ear2* (*Nr2f6*), and *Treml4*, genes associated with neuroblastoma staging and poor survival. *FABP4*+ macrophages, enriched in *MYCN*-amplified neuroblastoma relapse, have been implicated in enhancing tumor cell migration, invasion, and growth.

**Conclusions:**

Our data reveals the distant influence of primary tumors on bone marrow immune composition and signaling. These insights into bone marrow immune remodeling highlight FABP4+ macrophages and semaphorin signaling as potential immunotherapeutic targets to disrupt neuroblastoma progression.

**Figure:**
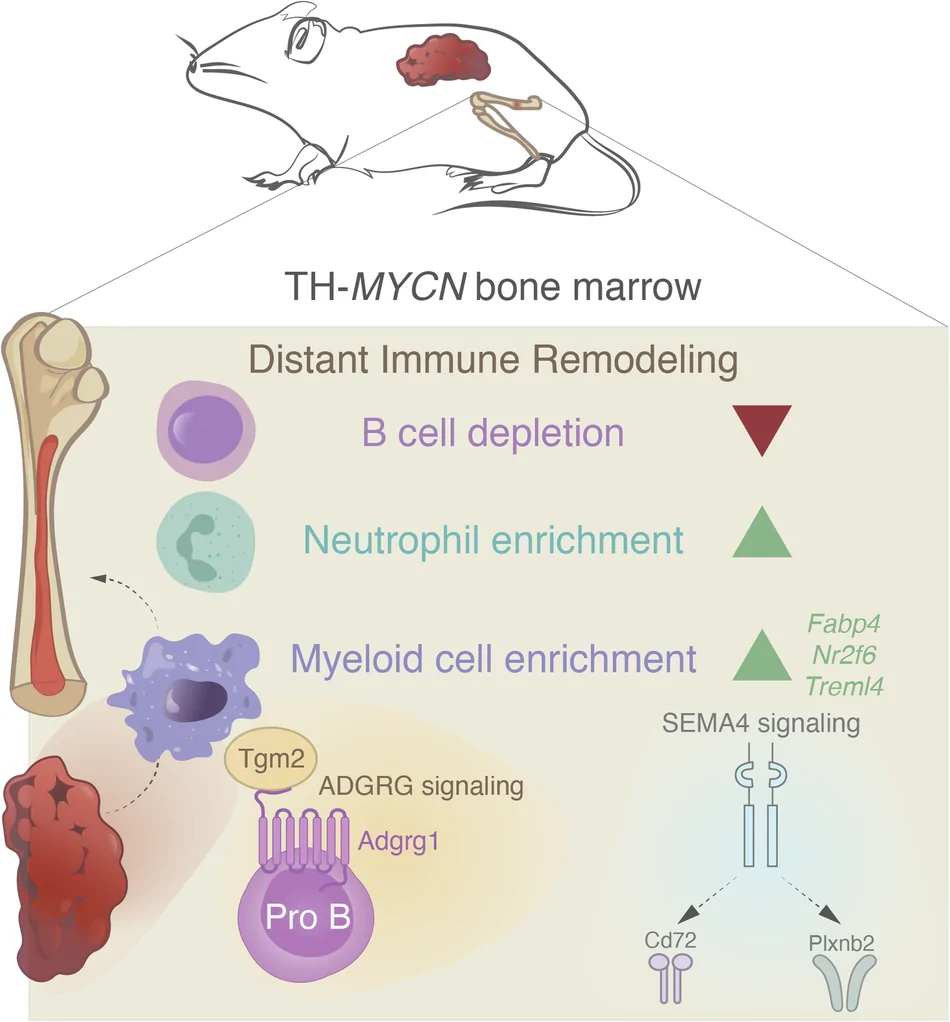

## Introduction

Neuroblastoma is a highly metastatic disease, and the majority of patients present with metastasis at diagnosis [1]. Bone marrow and bone are the most common metastatic sites, accounting for over 70% of neuroblastoma patients with metastasis and worse overall survival rates [1, 2]. Single cell transcriptomics of human neuroblastoma bone marrow metastases have mapped the immunosuppressive metastatic microenvironment [3–5]. The metastatic bone marrow niche exhibited increased myelocytes/immature neutrophils, neutrophils, and cytotoxic T cells, while there was a depletion of developing B cell [3–5]. In our recent study, single-cell RNA sequencing (scRNA-seq) and flow cytometry analyses confirmed a significant decrease in B cell populations in neuroblastoma patients with established bone marrow metastases [3]. Additionally, ligand-receptor interaction analysis revealed potential immunoregulatory interaction CD24-SIGLEC10, expressed by tumor cells and macrophages, respectively [3].

Amplification of *MYCN* oncogene is found in approximately 20% of neuroblastoma patients and is a risk-stratifying prognostic marker, associated with aggressive disease progression [6, 7]. Overexpression of *MYCN* under the tyrosine hydroxylase (TH) promoter in mice, gives rise to tumors exclusively in the sympathoadrenal system, mimicking human *MYCN*-amplified neuroblastoma features [8, 9]. A comparative study of the immune cells in TH-*MYCN* tumors and human neuroblastoma biopsies identified a shared immunosuppressive tumor microenvironment, including low T-cell content, diverse macrophage subtypes and distinct cells expressing myeloid-derived suppressor cell (MDSC)-related genes [10].

Although TH-*MYCN* mice exhibit limited bone marrow metastasis (<5% incidence) [8], efforts have also been devoted to the development of immunocompetent bone marrow-metastatic models, including a caspase-8-deficient variant [11] and a chemo-resistant model (Th-*MYCN*^CPM32^), which better mimic the bone metastases observed in relapsed neuroblastoma patients [12]. Despite the rarity of bone marrow infiltration in the TH-*MYCN* mice, this mouse model provides a valuable tool for studying the impact of a distant primary tumor on the bone marrow composition and immunoregulatory remodeling that shape the bone marrow microenvironment of *MYCN*-driven high-risk neuroblastoma.

Here, we sequence matched TH-*MYCN* tumor and bone marrow samples to evaluate the distant influence of the primary tumor on the bone marrow. Our recent study explored TH-*MYCN* tumors and novel organoid models [9]. In this study, however, we shift our focus to the bone marrow. We conduct a comparative analysis of the bone marrow transcriptomes from wild-type (WT) and tumor-bearing TH-*MYCN* mice, exploring changes in cellular composition and identifying distinct intercellular communication pathways that may contribute to an immunosuppressive milieu in the bone marrow.

## Methods

The TH-*MYCN* transgenic mice used in this study are the same as described in ref. [9]. Five TH-*MYCN* transgenic mice (N16 backcross to the 129 × 1/SvJ background) were included, comprising three homozygous mice (ages 37, 40 and 41 days) and two hemizygous mice (ages 58 and 97 days). The control group consisted of three WT mice 129 × 1/SvJ background (ages 90, 46 and 48 days).

After euthanasia, the femurs, tibia and tumors were collected for mechanical and enzymatic dissociation, and FACS enrichment of viable single cells. Sorted cells were used for library preparation with 10x Genomics Chromium 3’ v2 kit, and sequenced on the Illumina NextSeq 550 platform. The sequencing coverage and quality statistics for each sample are summarized in Supplementary Table S1.

Raw sequencing data were aligned to a modified GRCm38 reference using Cellranger v6.1.2 with an appended MYCN chromosome for transgenic samples. Quality filtering excluded cells based on UMI and mitochondrial content thresholds, with specific criteria detailed in Supplemental Methods. Processed data were integrated, normalized, and analyzed for highly variable genes and clustering using the Seurat v4.9.9 pipeline. Differentially expressed genes were identified (FindAllMarkers), clusters annotated by reference to marker genes (Tables S2–S4), and cell-cell interaction analysis performed using CellChat v1.5.0.

Immunostaining was conducted on paraffin-embedded, decalcified bone and tumor tissue sections. To validate the specificity of the immunohistochemistry staining, we included appropriate positive and negative controls. CD209/DC-SIGN staining was confirmed in spleen as a positive control, while a no-primary-antibody control was included for bone tissue (Fig. S1). Positive control staining was also performed for CEACAM1 and PLXNB2 in colon cancer tissue, and for SEMA4D in lymph node tissue (Fig. S2). Quantitative image analysis was conducted using a ZEISS Axioscan 7 and QuPath v0.5.1. Immunofluorescence was performed by antigen retrieval, followed by staining with primary (Table S5) and secondary (Table S6) antibodies, imaging on a Zeiss LSM980-Airy confocal microscope, and analysis with ImarisViewer v10.1.0. Statistical analyses were performed using GraphPad Prism v10.4.1 as specified in the figure legends. Further technical details are provided in the Supplemental Methods.

## Results

### Single-cell transcriptomic profiling of immune microenvironment in bone marrow and primary tumor from TH-*MYCN* mice

**Fig. 1:**
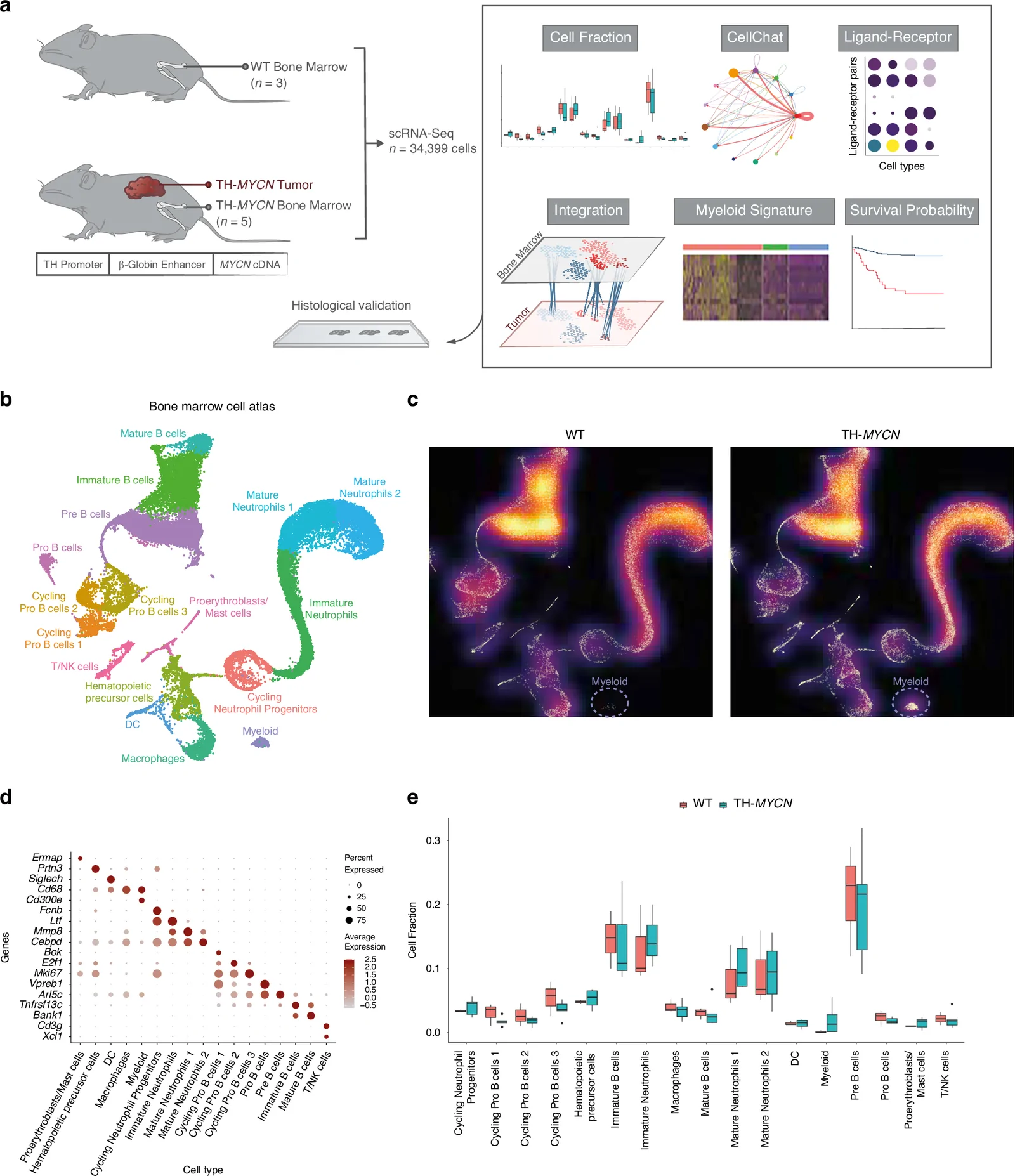
The single-cell landscape of wild-type and TH-*MYCN* bone marrow. **A** Schematic overview of samples and scRNA-seq data analysis. **B** UMAP embedding of bone marrow cells (34,399 cells) derived from five TH-*MYCN* mice and three wild-type mice. Major cell populations are colored and labeled. **C** Density plot comparing major cell populations between bone marrow samples of wild-type (WT) and TH-*MYCN* mice. Brighter colors correspond to denser regions. **D** Dot plot of cluster-specific marker genes in bone marrow cells. **E** Cell fraction analysis of relative abundance of different cell populations between bone marrow samples of WT and TH-*MYCN* mice.

To explore the cellular and transcriptional changes in bone marrow of tumor-bearing mice, viable non-erythroid bone marrow cells were profiled by scRNA-seq, from five matched TH-*MYCN* mice and additional three WT mice (Table S1). This matched sampling enables direct comparison of the immune microenvironment of tumors and bone marrow (Fig. 1A). Bone marrow sequencing yielded a total of 34,399 cells, while tumor sequencing yielded 42,332 cells (Table S1-3). Unbiased clustering of integrated bone marrow samples revealed a comprehensive repertoire of 17 immune clusters undergoing continuous hematopoiesis, originating from hematopoietic precursor cells that give rise to B cells, erythroid, granulocyte, and myeloid lineages (Table S2 and Fig. 1B, C). B cell development was apparent in the bone marrow, with seven subclusters of B cells: pro B cells (*Vpreb1*), cycling pro B cells 1 (*Bok*), cycling pro B cells 2 (*E2f1*), cycling pro B cells 3 (*Mki67*), pre B cells (*Arl5c*), immature B cells (*Tnfrsf13c*) and mature B cells (*Bank1*) (Fig. 1D) [13]. The hematopoietic precursor cell cluster (*Prtn3*) was directly connected to dendritic cells (DC) (*Siglech*) and macrophages (*Cd68*), as well as granulocyte lineages including proerythroblasts (*Ermap*) and neutrophil development: cycling neutrophil progenitors (*Fcnb*), immature neutrophils (*Ltf*), mature neutrophils 1 (*Mmp8*) and mature neutrophils 2 (*Cebpd*) (Fig. 1B–D) [14, 15].

Interestingly, a distinct myeloid cell population expressing *Cd300e* was disconnected from other myeloid populations in the UMAP embedding (Fig. 1B). Cd300e has been described as an immune-activating receptor [16], expressed by monocytes and tissue macrophages [17]. However, it has also been shown to negatively regulate T cell activation by impairing STAT1 pathway activated by IFN-γ [17]. Notably, upregulated expression of CD300E across multiple cancers is associated with poor patient survival [18]. Moreover, a study characterizing the immune landscape of TH-*MYCN* tumors revealed a subcluster of macrophages expressing *Cd300e* amongst other markers, including *Pecam1* and *Cd274* (PD-L1) [10]. The subclusters of *Cd68* + *Cd300e*+ myeloid cells in the bone marrow may correspond to tumor-associated macrophages, which we explored below.

The presence of abdominal tumors indicated marked alterations in the immune cell composition of the distant bone marrow (Fig. 1C). While these changes were not statistically significant, notable trends were observed (Fig. 1E). Compared to WT, the bone marrow of TH-*MYCN* mice had an increased proportion of a *Cd300e*+ myeloid cluster. Additionally, the bone marrow of TH-*MYCN* mice indicated an increase in neutrophil populations, and a reduction in developing B cell clusters. These changes are consistent with our scRNA-seq study of human neuroblastoma bone marrow metastatic microenvironment involving neutrophil enrichment and a reduction in B cell populations [3]. This suggests that the immunological environment in the bone marrow of tumor-bearing TH-*MYCN* mice may be undergoing remodeling, mirroring the changes observed in human neuroblastoma bone marrow metastasis.

Next, we explored the presence of disseminated tumor cells in the bone marrow. Immunohistochemistry showed no detectable N-MYC or PHOX2B positive tumor cells (Fig. S3). In addition, we examined gene expression of oncogenic neuroblastoma transcription factors *MYCN*, *Phox2b*, *Phox2a*, *Isl1*, and neurofilament markers *Nefl*, *Nefm*, and *Nefh* across bone marrow samples from TH-*MYCN* and WT mice (Fig. S4). We detected very few *MYCN* and *Nefh* positive cells in one homozygous TH-*MYCN* bone marrow sample, however, no distinct tumor cell population was identified in the bone marrow.

scRNA-seq of matched TH-*MYCN* tumors (Table S1) revealed 16 major cell clusters, which were present in all samples (Fig. S5A–C). A total of 35846 cells made up the tumor cell compartment, 5918 cells were assigned to the immune compartment and 568 stromal cells. Cell cluster annotations were determined based on the expression of key marker genes (Table S3, 4, Fig. S5A–C). The stromal cells comprised endothelial cells (expressing *Cdh5*, *Emcn, Gpihbp1*) and mesenchymal cells (*Mgp*, *Col1a1, Prrx1*, *Pdgfrb*, *Dcn*) (Fig. S5D, E). Lymphoid populations included B cells (*Cd79a*, *Ms4a1*, *Ighd*), T cells (*Cd3d, Trac*, *Trbc2*) and a minor subpopulation of NK cells (*Xcl1*, *Ncr1*, *Klre1*) (Fig. S5D–F). The immune compartment also comprised myeloid lineage cells (*Lyz2*, *Fcer1g*, *Clec4a3*), with macrophages (*Cd68*) constituting the majority of this population (Fig. S5D–E). The remaining leukocyte populations included neutrophils (*S100a9*, *S100a8*), myeloid dendritic cells (mDC) (*Syngr2*, *Ahr*), as well as plasmacytoid dendritic cells (pDC) (*Clec10a*, *Siglech*) (Fig. S5D, E). To further examine the neutrophil-associated signature in TH-*MYCN* tumors, we analyzed expression of *Padi4*, *Ngp*, and *Mpo* in homozygous and hemizygous tumors. *Ngp* and *Padi4* were expressed in the neutrophil population in both tumor groups, whereas *Mpo* showed limited expression (Figs. S6, S7).

### Context specific immunoregulatory interactions in the bone marrow of TH-*MYCN* mice

**Fig. 2:**
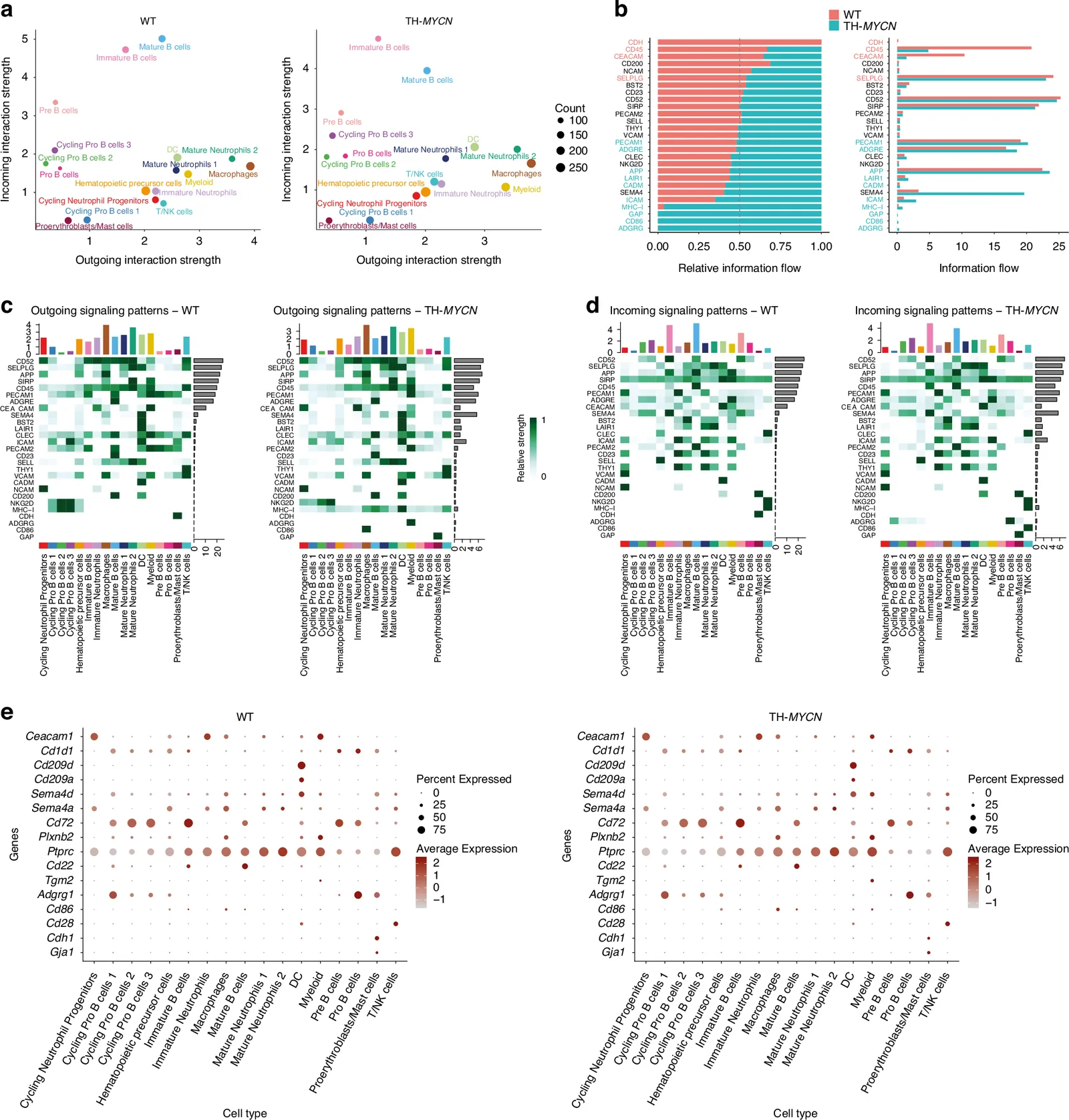
Identification of sophisticated communication network of TH-*MYCN* bone marrow microenvironment. **A** Scatter plots of outgoing and incoming interaction strength of each cell population in the bone marrow of WT and TH-*MYCN* mice. **B** Bar plot showing the relative information flow within inferred networks and ranked based on the differences of overall information flow within these networks between the bone marrow of WT and TH-*MYCN* mice. **C** Heatmap of outgoing signaling strength of each signaling pathway in each cell population in bone marrows samples from WT (Left) and TH-*MYCN* (Right) mice. **D** Heatmap of incoming signaling strength of each signaling pathway in each cell population in bone marrows samples from WT (Left) and TH-*MYCN* (Right) mice. **E** Dot plot of gene expression of significant ligand-receptor pairs contributing to CEACAM, SEMA4, CD45, ADGRG, CD86, CDH and GAP signaling pathways.

Next, we compared communication pathways in the bone marrow between WT and TH-*MYCN* mice, using CellChat analysis. Evaluation of major outgoing and incoming signals across cell populations showed that macrophages, mature neutrophils and myeloid cells were the dominant signaling sources (Fig. 2A). In WT bone marrow, mature B cells were the main signaling targets, while immature B cells were the top targets in TH-*MYCN* bone marrow samples (Fig. 2A). To distinguish between conserved and context-specific signaling pathways, we compared the overall information flow for each pathway, calculated as the cumulative communication probability across all cell populations in each condition. As illustrated in Fig. 2B, most signaling pathways were shared between both WT and TH-*MYCN* mice. Relative information flow showed that only CDH pathway was unique to WT, however, the total information flow was low from only proerythroblasts/mast cells (Fig. 2B–D). On the other hand, GAP, CD86, and ADGRG pathways were uniquely observed in the bone marrow of TH-*MYCN* mice (Fig. 2B–D). All three pathways are notable due to their established roles in immune regulation and bone/bone marrow metastasis [19–21].

Major differences in information flow revealed context-specific pathways in each sample group. Of note, CD45 and CEACAM pathways were more prominent in WT bone marrow, whereas SEMA4 signaling pathway predominated in TH-*MYCN* bone marrow (Fig. 2B). Ligand-receptor pairs that contributed to the CD45 signaling pathway were Ptprc-Cd22 (Fig. S8A), of which *Cd22* was expressed by immature and mature B cells (Fig. 2E) that were in higher abundance in the WT bone marrow. High expression of both *PTPRC* and *CD22* were associated with a better clinical outcome in the SEQC-489 neuroblastoma patient cohort (Figs. S8–S9), possibly due to higher levels of lymphocyte infiltration. In both *MYCN*-amplified and non-amplified groups, high *PTPRC* expression was associated with improved overall survival (Fig. S9A), and *PTPRC* expression was negatively correlated with INSS stage in the SEQC cohort (Fig. S9B). Evaluation of the contribution of ligand-receptor pairs to the CEACAM signaling pathway revealed Ceacam1-Ceacam1, Ceacam1-Cd209d, Ceacam1-Cd1d1 and Cd209a-Ceacam1 (Fig. S8A). High expression of *CD1D* was also significantly associated with improved prognosis in the SEQC-489 neuroblastoma patient cohort (Fig. S8C). DCs express the C-type lectin dendritic cell–specific intercellular adhesion molecule-3–grabbing nonintegrin (DC-SIGN/*Cd209*) (Fig. 2E), which facilitates the recognition of pathogens or tumor cells [22, 23]. Both *Ceacam1* and *Cd209* are predicted ligands and receptors in the CEACAM signaling pathway (Fig. S10A). Histologically we confirmed the widespread protein expression of CEACAM1 and DC-SIGN in WT bone marrow (Fig. S10B). Since high *CEACAM1* expression has been associated with immune cell infiltration and improved prognosis in clear cell renal cell carcinoma (ccRCC), we explored the expression of both *CEACAM1* and *CD209* in tumors from the SEQC-489 neuroblastoma patient cohort. Low expression of *CEACAM1* and *CD209* was associated with poor survival probability (Fig. S10C, D), and both genes showed significant negative correlation with INSS stage (Fig. S11).

The ligand-receptor contributors within the SEMA4 signaling pathway were Sema4d-Cd72, Sema4a-Plxnb2 and Sema4d-Plxnb2 (Fig. S8A). The latter interaction, Sema4d-Plxnb2, has been reported in primary neuroblastoma patient tumors, specifically interactions between tumor cells and T/NK cells, as well as between myeloid cells and T/NK cells [24]. Similarly, this predicted interaction was detected between macrophages and myeloid cells with T/NK cells in TH-*MYCN* bone marrow, as well as distinct autocrine signaling within the myeloid lineage (Fig. S12A). Histologically, SEMA4D and PLXNB2 was observed in the growth plates of TH-*MYCN* femurs (Fig. S12B). Immunohistochemical evaluation revealed higher proportions of SEMA4D+ cells in TH-*MYCN* bone as compared to WT bone (Fig. S12C).

**Fig. 3:**
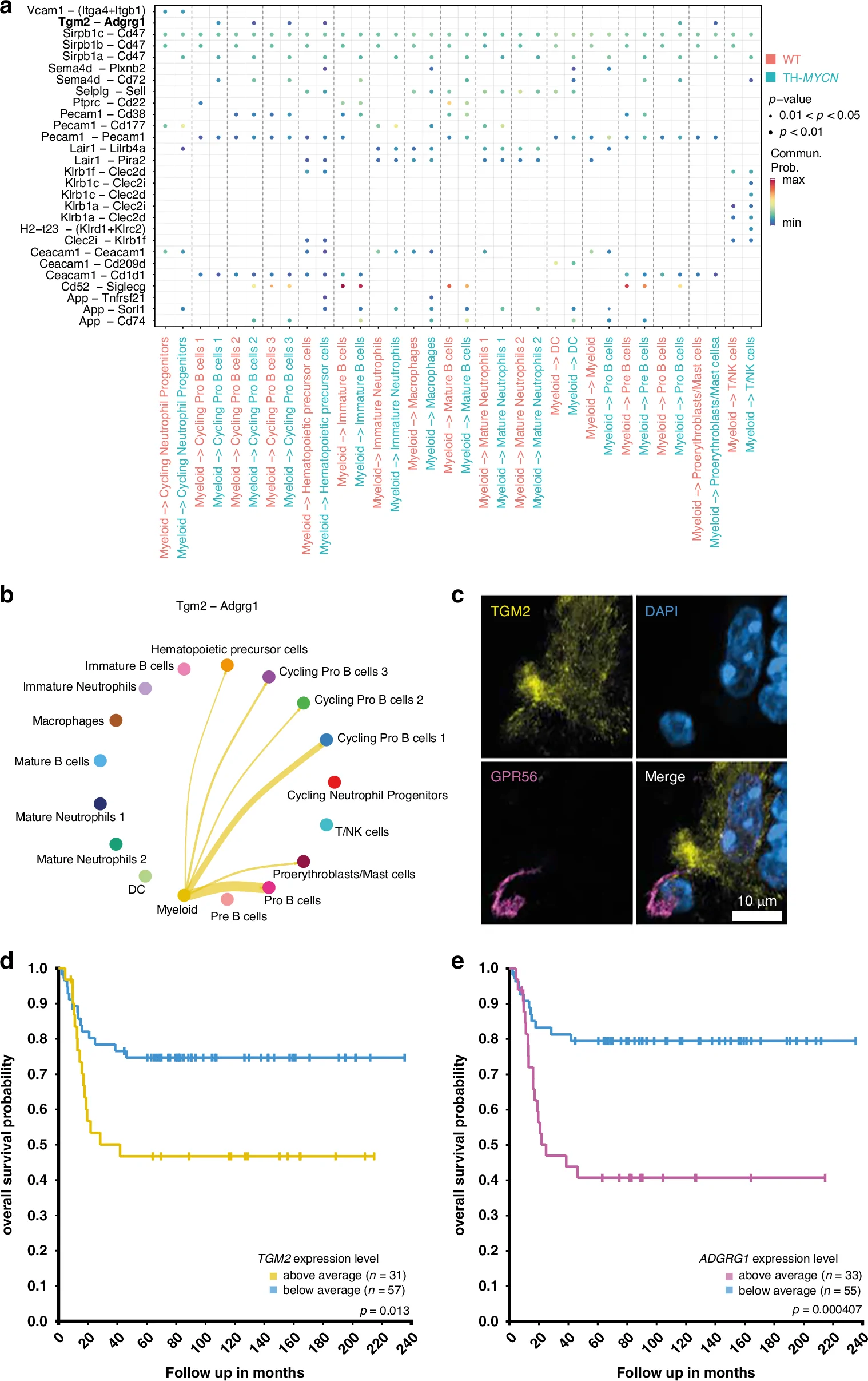
TH-*MYCN* bone marrow myeloid cells contribute to Tgm2 - Adgrg1 signaling. **A** Bubble plot comparison of the significant predicted ligand-receptor pairs between WT and TH-*MYCN* bone marrow samples, which contribute to the signaling from myeloid cells. Communication probabilities are shown by dot color, while calculated p-values are represented by dot size. The communication probability is 0 when there is an empty space. The one-sided permutation test is used to calculate *p*-values. **B** Circle plot of the inferred Tgm2-Adgrg1 signaling network. The line thickness indicates the communication probability. **C** Immunostaining for TGM2 (yellow) and GPR56/ADGRG1 (magenta) in TH-*MYCN* bone. **D** Kaplan–Meier curve showing the overall survival in 88 patients with neuroblastoma (Versteeg, GEO: GSE16476 88/122), separated by average *TGM2* expression. Yellow, *TGM2* above average (high); blue, *TGM2* below average (low). **E** Kaplan–Meier curve showing the overall survival in 88 patients with neuroblastoma (Versteeg, GEO: GSE16476 88/122), separated by average *ADGRG1* expression. Magenta, *ADGRG1* above average (high); blue, *ADGRG1* below average (low).

ADGRG signaling pathway was exclusively detected in TH-*MYCN* bone marrow. The ADGRG signaling source was the distinct myeloid population (Fig. 2C). Comparative ligand-receptor analysis which contributed to outgoing signaling from the myeloid population revealed significant Tgm2-Adgrg1 interaction solely in TH-*MYCN* bone marrow (Fig. 3A). Inferred Tgm2-Adgrg1 signaling network showed communication from myeloid to pro B cell populations (Fig. 3B). While the abundance of both the myeloid and pro B cell populations were low, TGM2 in proximity to GPR56/ADGRG1 was detected by immunostaining (Fig. 3C). Furthermore, high mRNA expression of *TGM2* and *GPR56*/*ADGRG1* was associated with poor prognosis in three neuroblastoma patient cohorts (Fig. 3D, E, Fig. S12D–E).

### Distinct myeloid cell signatures in the bone marrow microenvironment of neuroblastoma

Since myeloid cells, including MDSCs and monocytes, play a key role in neuroblastoma bone marrow metastasis [4, 5], we conducted a detailed analysis of distinct myeloid populations in TH-*MYCN* bone marrow. Previous research on TH-*MYCN* tumors identified three macrophage subsets [10], so we aligned immune cells from TH-*MYCN* bone marrow and matched tumors (Fig. S13A). Immune cell lineages were compared between the primary tumor and bone marrow (Fig. S13B), revealing major overlapping populations such as T cells, NK cells, mature B cells, mature neutrophils 2, DCs, macrophages, and myeloid cells (Fig. S13C).

**Fig. 4:**
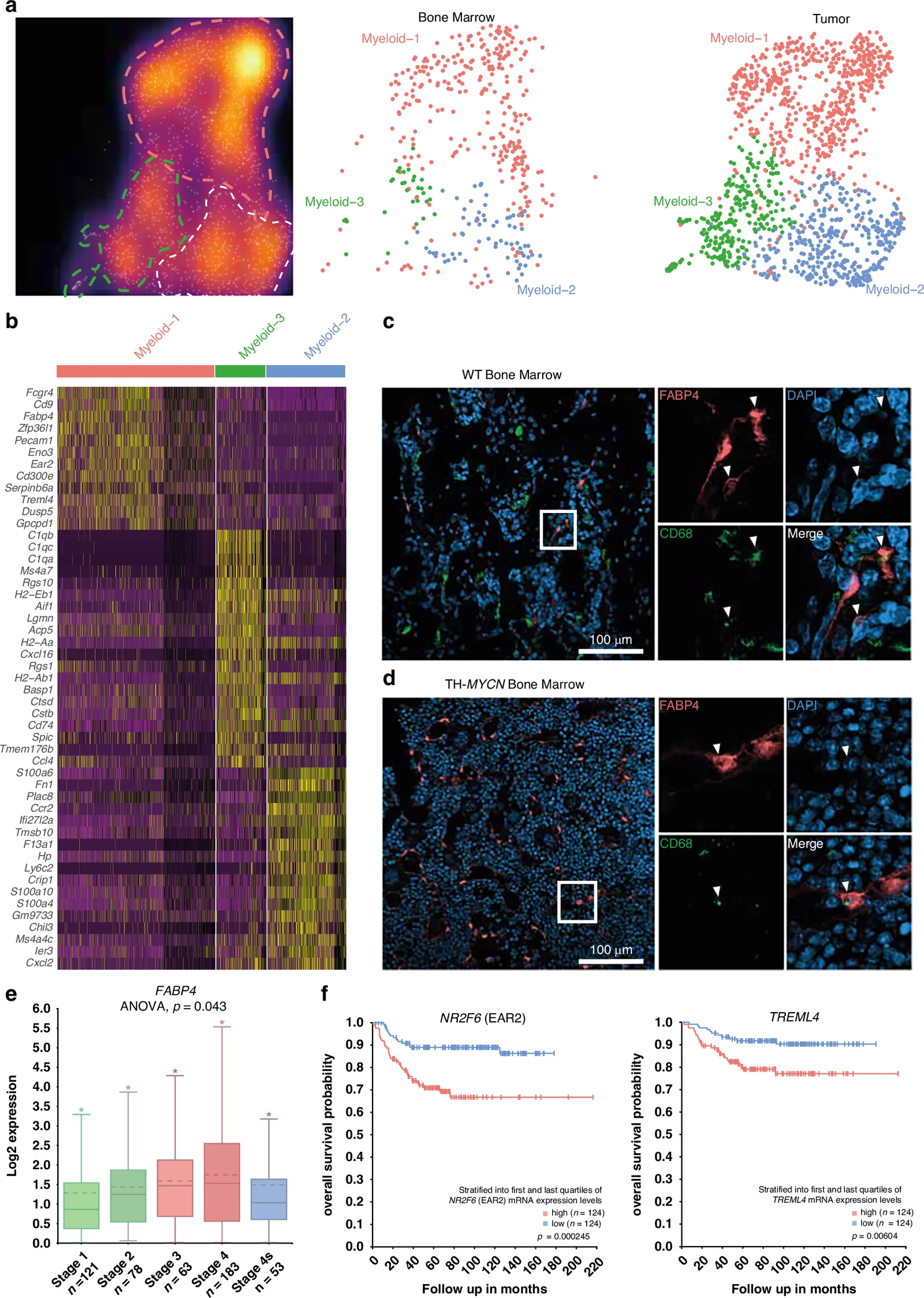
Enriched myeloid signature in the bone marrow of tumor-bearing mice. **A** Density plot of myeloid subclusters in TH-*MYCN* bone marrow (Left), in which brighter colors correspond to denser regions. UMAP embedding demonstrating myeloid subclusters in TH-*MYCN* bone marrow (Middle) and matched tumors (Right). **B** Heatmap showing expression of top differentially expressed genes (rows) across the cells (columns) in myeloid subclusters (top color bar, as in annotated UMAP on Fig. A). **C** Immunostaining for FABP4 (magenta) and CD68 (green) in WT bone marrow. **D** Immunostaining for FABP4 (magenta) and CD68 (green) in TH-*MYCN* bone marrow. White arrows indicate double-positive cells. **E** *FABP4* expression in patients with neuroblastoma from the SEQC cohort (*n *= 498, GEO: GSE49711) stratified according to the INSS stages. **F** Kaplan–Meier curve showing the overall survival in the SEQC cohort, stratified into the first and last quartiles based on the *NR2F6* (EAR2), or *TREML4* mRNA expression levels.

Focusing on the myeloid cluster specifically enriched in the bone marrow of TH-*MYCN* mice, we analyzed the integrated myeloid populations (Fig. S13D). Subcluster analysis identified three major macrophage gene signatures, *Pecam1* + , *Ccr2* + , and *Apoe*+ macrophages (Fig. S13E, F), mirroring subsets previously described in TH-*MYCN* tumors [10]. The Pecam1+ macrophage subset, marked by *Cd300e* expression, was annotated as Myeloid-1 (Fig. 4A, B). Myeloid-1 was the dominant myeloid subcluster in bone marrow (Fig. 4A) and displayed a distinct gene expression profile: *Pecam1*, *Cd300e*, *Fcgr4*, *Cd9*, *Fabp4*, *Treml4* (Fig. 4B), potentially representing a tumor-associated macrophage signature [16, 25–29]. *FABP4+* macrophages are exclusively enriched in biopsies from neuroblastoma patients with *MYCN*-amplification, specifically in samples collected at relapse [10]. We detected a higher abundance of FABP4+ macrophages in TH-*MYCN* bone compared to WT bone (Fig. 4C, D). This population of macrophages may be derived from the PECAM1+ macrophage subpopulation detected in TH-*MYCN* tumors (Fig. S13G) [10]. Notably, *FABP4* expression increased with INSS stage in the SEQC-489 neuroblastoma patient cohort (Fig. 4E). High expression of *EAR2* (*NR2F6*) and *TREML4*, were associated with worse overall patient survival outcomes (Fig. 4F) and in non-*MYCN*-amplified group (Fig. S14A). *NR2F6* expression was also significantly associated with INSS stage (Fig. S14B).

To further explore the clinical implications of the distinct myeloid gene expression patterns, we mapped *CD300E* and *PECAM1* expression onto human neuroblastoma bone marrow metastases from our previous study (Fig. S15A, B) [3]. Feature plots reveal their distinct expression in macrophages in metastatic samples (Fig. S15B). Inhibitory receptor *SIGLEC10* was amongst the differentially expressed genes in this cluster of macrophages (Fig. S15C-E). We have previously shown CD24-SIGLEC10 ligand-receptor interactions between tumor and macrophages in both primary neuroblastoma and bone marrow metastasis [3, 30], which may promote immune evasion [31].

## Discussion

In this study, we investigated the immune landscape of the bone marrow in TH-*MYCN* tumor-bearing mice through single-cell transcriptomic profiling. We observed notable trends in immune cell composition, including an increase in neutrophils and Cd300e+ myeloid cells, along with a reduction in developing B cell populations in TH-*MYCN* mice. While these shifts were not statistically significant, they suggest distant immune remodeling within the bone marrow of tumor-bearing mice that mirror changes previously reported in human neuroblastoma bone marrow metastases [3–5].

Our results align with existing literature on neuroblastoma bone marrow metastasis, where neutrophil enrichment and B cell depletion were also observed [3]. In both human neuroblastoma and mouse models, immune suppression appears to play a crucial role in fostering both the primary tumor microenvironment and metastatic niche [3–5, 10]. A shared immunosuppressive tumor microenvironment has been shown in TH-*MYCN* and human neuroblastomas, characterized by exhausted T cells and an abundance of MDSCs [10]. The observed trends in immune cell composition in our study further emphasize this immunosuppressive milieu, which may enable tumor progression and invasion.

The identification of distinct intercellular communication pathways provides insights into the molecular processes that condition the bone marrow microenvironment in response to a distant primary tumor. We detected GAP, CD86, and ADGRG signaling pathways which were unique to the TH-*MYCN* bone marrow. The CD86-CD28 co-stimulation axis supports regulatory T cell (Treg) expansion under high CTLA-4 levels [20]. ADGRG1, expressed by breast cancer cells, plays a pivotal role in promoting bone metastasis formation [21]. Additionally, within the enriched SEMA4 pathway, the Sema4D-Plexin-B2 axis is known to be involved in endothelial cell migration and angiogenesis, which enhances vascularization to support tumor growth [32, 33]. On the other hand, the Sema4D-Cd72 interaction is known to modulate immune responses particularly in the context of B cell activation and regulation [34]. While we did not retrieve bone cells such as osteoclasts and osteoblasts, Sema4D is known to play a significant role in bone remodeling, through its association with RANKL-activated osteoclasts [35]. Sema4D not only contributes to the regulation of bone remodeling but also facilitates the establishment of a favorable microenvironment for metastatic cancer cells in bone tissue [36].

A distinct population of myeloid cells was identified in the bone marrow of TH-*MYCN* mice but absent in WT mice. Joint alignment of this myeloid population with matched TH-*MYCN* tumors revealed similarities with a previously described Pecam1+ macrophage subcluster [10], and enrichment of *Fabp4*, *Ear2* and *Treml4* genes associated with poor prognosis. Notably, orphan nuclear receptor Ear2 (Nr2f6) has been proposed as an immune checkpoint target for cancer immunotherapy [37, 38]. Importantly, FABP4 in macrophages has been shown to promote neuroblastoma cell migration, invasion, and tumor growth, which may occur through the deactivation of the NF-κB-IL1α pathway by ubiquitinating ATPB [39]. FABP4 stands out as a key tumor-promoting molecule across multiple cancer types and presents a promising therapeutic target for cancer treatment [40].

## Supplementary information

Supplementary Figures

Supplemental Methods

Supplementary Figure Legend

Supplementary Tables

## Data Availability

The source code for this study will be available at Github repository: https://github.com/hiraksarkar/neuroblastoma_analysis. WT and TH-*MYCN* bone marrow raw sequencing data and processed data in this paper will be available under in Gene Expression Omnibus. TH-*MYCN* tumor data is publicly available under the accession no. GSE280892. The human neuroblastoma bone marrow data is available under the accession no. GSE220946 and Github repository: https://github.com/shenglinmei/NB.bone.Met (commit ID: 27b633c). Additionally, neuroblastoma bulk RNA-Seq data for survival analyses were retrieved from GSE49711. Other data that support the findings of this study are available from the corresponding author upon request.

## References

[1] Liu S, Yin W, Lin Y, Huang S, Xue S, Sun G, et al. Metastasis pattern and prognosis in children with neuroblastoma. World J Surg Oncol. 2023;21:130. 10.1186/S12957-023-03011-Y. 37046344 PMC10091559

[2] Merugu S, Chen L, Gavens E, Gabra H, Brougham M, Makin G, et al. Detection of circulating and disseminated neuroblastoma cells using the imagestream flow cytometer for use as predictive and pharmacodynamic biomarkers. Clin Cancer Res. 2020;26:122–34. 10.1158/1078-0432.CCR-19-0656/357957/P/DETECTION-OF-CIRCULATING-AND-DISSEMINATED. 31767563

[3] Mei S, Alchahin AM, Embaie BT, Gavriliuc IM, Verhoeven BM, Zhao T, et al. Single-cell analyses of metastatic bone marrow in human neuroblastoma reveals microenvironmental remodeling and metastatic signature. JCI Insight. 2024;9:e173337. 10.1172/JCI.INSIGHT.173337. 38358826 PMC10972621

[4] Fetahu IS, Esser-Skala W, Dnyansagar R, Sindelar S, Rifatbegovic F, Bileck A, et al. Single-cell transcriptomics and epigenomics unravel the role of monocytes in neuroblastoma bone marrow metastasis. Nat Commun. 2023;14:3620. 10.1038/s41467-023-39210-0. 37365178 PMC10293285

[5] Lazic D, Kromp F, Rifatbegovic F, Repiscak P, Kirr M, Mivalt F, et al. Landscape of bone marrow metastasis in human neuroblastoma unraveled by transcriptomics and deep multiplex imaging. Cancers (Basel). 2021;13:4311. 10.3390/CANCERS13174311/S1. 34503120 PMC8431445

[6] Schwab M. MYCN in neuronal tumours. Cancer Lett. 2004;204:179–87. 10.1016/S0304-3835(03)00454-3. 15013217

[7] Matthay KK, Maris JM, Schleiermacher G, Nakagawara A, Mackall CL, Diller L, et al. Neuroblastoma. Nat Rev Dis Prim. 2016;2:16078. 10.1038/nrdp.2016.78. 27830764

[8] Weiss WA, Aldape K, Mohapatra G, Feuerstein BG, Bishop JM. Targeted expression of MYCN causes neuroblastoma in transgenic mice. EMBO J. 1997;16:2985–95. 10.1093/EMBOJ/16.11.2985. 9214616 PMC1169917

[9] Embaie BT, Sarkar H, Alchahin AM, Otte J, Olsen TK, Tümmler C, et al. Comparative single-cell transcriptomics of human neuroblastoma and preclinical models reveals conservation of an adrenergic cell state. Cancer Res. 2025;85:1015–34. 10.1158/0008-5472.CAN-24-1507. 39808065 PMC11907193

[10] Costa A, Thirant C, Kramdi A, Pierre-Eugène C, Louis-Brennetot C, Blanchard O, et al. Single-cell transcriptomics reveals shared immunosuppressive landscapes of mouse and human neuroblastoma. J Immunother Cancer. 2022;10:4807. 10.1136/JITC-2022-004807. PMC9362821 36054452

[11] Teitz T, Inoue M, Valentine MB, Zhu K, Rehg JE, Zhao W, et al. Th-MYCN mice with caspase-8 deficiency develop advanced neuroblastoma with bone marrow metastasis. Cancer Res. 2013;73:4086–97. 10.1158/0008-5472.CAN-12-2681. 23536557 PMC3702642

[12] Yogev O, Almeida GS, Barker KT, George SL, Kwok C, Campbell J, et al. In vivo modeling of chemoresistant neuroblastoma provides new insights into chemorefractory disease and metastasis. Cancer Res. 2019;79:5382–93. 10.1158/0008-5472.CAN-18-2759. 31405846

[13] Lee RD, Munro SA, Knutson TP, LaRue RS, Heltemes-Harris LM, Farrar MA. Single-cell analysis identifies dynamic gene expression networks that govern B cell development and transformation. Nat Commun. 2021;12:6843. 10.1038/s41467-021-27232-5. 34824268 PMC8617197

[14] Evrard M, Kwok IWH, Chong SZ, Teng KWW, Becht E, Chen J, et al. Developmental analysis of bone marrow neutrophils reveals populations specialized in expansion, trafficking, and effector functions. Immunity. 2018;48:364–379.e8. 10.1016/j.immuni.2018.02.002. 29466759

[15] Kwok I, Becht E, Xia Y, Ng M, Teh YC, Tan L, et al. Combinatorial single-cell analyses of granulocyte-monocyte progenitor heterogeneity reveals an early uni-potent neutrophil progenitor. Immunity. 2020;53:303–318.e5. 10.1016/j.immuni.2020.06.005. 32579887

[16] Isobe M, Izawa K, Sugiuchi M, Sakanishi T, Kaitani A, Takamori A, et al. The CD300e molecule in mice is an immune-activating receptor. J Biol Chem. 2018;293:3793–805. 10.1074/jbc.RA117.000696. 29358324 PMC5846139

[17] Coletta S, Salvi V, Della Bella C, Bertocco A, Lonardi S, Trevellin E, et al. The immune receptor CD300e negatively regulates T cell activation by impairing the STAT1-dependent antigen presentation. Sci Rep. 2020;10:16501. 10.1038/s41598-020-73552-9. 33020563 PMC7536427

[18] Luo Z, Zhu J, Xu R, Wan R, He Y, Chen Y, et al. Exercise-downregulated CD300E acted as a negative prognostic implication and tumor-promoted role in pan-cancer. Front Immunol. 2024;15:1437068. 10.3389/FIMMU.2024.1437068. 39144140 PMC11321962

[19] Sinha S, Callow BW, Farfel AP, Roy S, Chen S, Masotti M, et al. Breast cancers that disseminate to bone marrow acquire aggressive phenotypes through CX43-related tumor-stroma tunnels. J Clin Investig. 2024;134:e170953. 10.1172/JCI170953. 39480488 PMC11645149

[20] Halliday N, Williams C, Kennedy A, Waters E, Pesenacker AM, Soskic B, et al. CD86 is a selective CD28 ligand supporting FoxP3+ regulatory T cell homeostasis in the presence of high levels of CTLA-4. Front Immunol. 2020;11:600000. 10.3389/fimmu.2020.600000. 33363541 PMC7753196

[21] Sasaki Sichiro, Zhang D, Iwabuchi S, Tanabe Y, Hashimoto S, Yamauchi A, et al. Crucial contribution of GPR56/ADGRG1, expressed by breast cancer cells, to bone metastasis formation. Cancer Sci. 2021;112:4883–93. 10.1111/CAS.15150. 34632664 PMC8645723

[22] Engering A, Geijtenbeek TBH, van Vliet SJ, Wijers M, van Liempt E, Demaurex N, et al. The dendritic cell-specific adhesion receptor DC-SIGN internalizes antigen for presentation to T cells. J Immunol. 2002;168:2118–26. 10.4049/JIMMUNOL.168.5.2118. 11859097

[23] Van Gisbergen KPJM, Aarnoudse CA, Meijer GA, Geijtenbeek TBH, Van Kooyk Y. Dendritic cells recognize tumor-specific glycosylation of carcinoembryonic antigen on colorectal cancer cells through dendritic cell–specific intercellular adhesion molecule-3–grabbing nonintegrin. Cancer Res. 2005;65:5935–44. 10.1158/0008-5472.CAN-04-4140. 15994972

[24] Wienke J, Visser LL, Kholosy WM, Keller KM, Barisa M, Poon E, et al. Integrative analysis of neuroblastoma by single-cell RNA sequencing identifies the NECTIN2-TIGIT axis as a target for immunotherapy. Cancer Cell. 2024;42:283–300.e8. 10.1016/J.CCELL.2023.12.008. 38181797 PMC10864003

[25] Hey YY, O’Neill TJ, O’Neill HC. A novel myeloid cell in murine spleen defined through gene profiling. J Cell Mol Med. 2019;23:5128–43. 10.1111/JCMM.14382. 31210415 PMC6653018

[26] Kitamura T, Doughty-Shenton D, Cassetta L, Fragkogianni S, Brownlie D, Kato Y, et al. Monocytes differentiate to immune suppressive precursors of metastasis-associated macrophages in mouse models of metastatic breast cancer. Front Immunol. 2018;8:2004. 10.3389/FIMMU.2017.02004. 29387063 PMC5776392

[27] Brosseau C, Colas L, Magnan A, Brouard S. CD9 tetraspanin: a new pathway for the regulation of inflammation?. Front Immunol. 2018;9:2316 10.3389/FIMMU.2018.02316. 30356731 PMC6189363

[28] Tian W, Zhang W, Zhang Y, Zhu T, Hua Y, Li H, et al. FABP4 promotes invasion and metastasis of colon cancer by regulating fatty acid transport. Cancer Cell Int. 2020;20:512. 10.1186/S12935-020-01582-4. 33088219 PMC7574203

[29] Guo D, Lin C, Lu Y, Guan H, Qi W, Zhang H, et al. FABP4 secreted by M1-polarized macrophages promotes synovitis and angiogenesis to exacerbate rheumatoid arthritis. Bone Res. 2022;10:45. 10.1038/S41413-022-00211-2. 35729106 PMC9213409

[30] Verhoeven BM, Mei S, Olsen TK, Gustafsson K, Valind A, Lindström A, et al. The immune cell atlas of human neuroblastoma. Cell Rep Med. 2022;3:100657. 10.1016/J.XCRM.2022.100657. 35688160 PMC9245004

[31] Barkal AA, Brewer RE, Markovic M, Kowarsky M, Barkal SA, Zaro BW, et al. CD24 signalling through macrophage Siglec-10 is a target for cancer immunotherapy. Nature. 2019;572:392–6. 10.1038/S41586-019-1456-0. 31367043 PMC6697206

[32] Ch’ng ES, Kumanogoh A. Roles of Sema4D and Plexin-B1 in tumor progression. Mol Cancer. 2010;9:251. 10.1186/1476-4598-9-251. 20858260 PMC2955613

[33] Basile JR, Castilho RM, Williams VP, Gutkind JS. Semaphorin 4D provides a link between axon guidance processes and tumor-induced angiogenesis. Proc Natl Acad Sci USA. 2006;103:9017–22. 10.1073/PNAS.0508825103. 16754882 PMC1482558

[34] Kumanogoh A, Shikina T, Watanabe C, Takegahara N, Suzuki K, Yamamoto M, et al. Requirement for CD100-CD72 interactions in fine-tuning of B-cell antigen receptor signaling and homeostatic maintenance of the B-cell compartment. Int Immunol. 2005;17:1277–82. 10.1093/intimm/dxh307. 16113236

[35] Ishii T, Ruiz-Torruella M, Yamamoto K, Yamaguchi T, Heidari A, Pierrelus R, et al. Locally secreted semaphorin 4D is engaged in both pathogenic bone resorption and retarded bone regeneration in a ligature-induced mouse model of periodontitis. Int J Mol Sci. 2022;23:5630. 10.3390/IJMS23105630. 35628440 PMC9148012

[36] Lontos K, Adamik J, Tsagianni A, Galson DL, Chirgwin JM, Suvannasankha A. The role of semaphorin 4D in bone remodeling and cancer metastasis. Front Endocrinol. 2018;9:322. 10.3389/FENDO.2018.00322. PMC6018527 29971044

[37] Miftah H, Naji O, Ssi SA, Ghouzlani A, Lakhdar A, Badou A. NR2F6, a new immune checkpoint that acts as a potential biomarker of immunosuppression and contributes to poor clinical outcome in human glioma. Front Immunol. 2023;14:1139268. 10.3389/FIMMU.2023.1139268. 37575237 PMC10419227

[38] Klepsch V, Siegmund K, Baier G. Emerging next-generation target for cancer immunotherapy research: the orphan nuclear receptor NR2F6. Cancers (Basel). 2021;13:2600. 10.3390/CANCERS13112600. 34073258 PMC8197903

[39] Miao L, Zhuo Z, Tang J, Huang X, Liu J, Wang HY, et al. FABP4 deactivates NF-κB-IL1α pathway by ubiquitinating ATPB in tumor-associated macrophages and promotes neuroblastoma progression. Clin Transl Med. 2021;11:e395. 10.1002/CTM2.395. 33931964 PMC8087928

[40] Sun N, Zhao X. Therapeutic implications of FABP4 in cancer: an emerging target to tackle cancer. Front Pharmacol. 2022;13:948610. 10.3389/FPHAR.2022.948610. 35899119 PMC9310032

