# Supplementary material for: Immune remodeling and myeloid signature in the bone marrow niche of MYCN-driven neuroblastoma
Source: BJC Rep. 2026 Sep 15;4:46. doi: 10.1038/s44276-026-00249-3 (PMC13578270; doi:10.1038/s44276-026-00249-3)
Supplement: Supplementary file 1 [file 44276_2026_249_MOESM1_ESM.pdf]

Figure S1

DC-SIGN (CD209) IHC
Positive Control Spleen

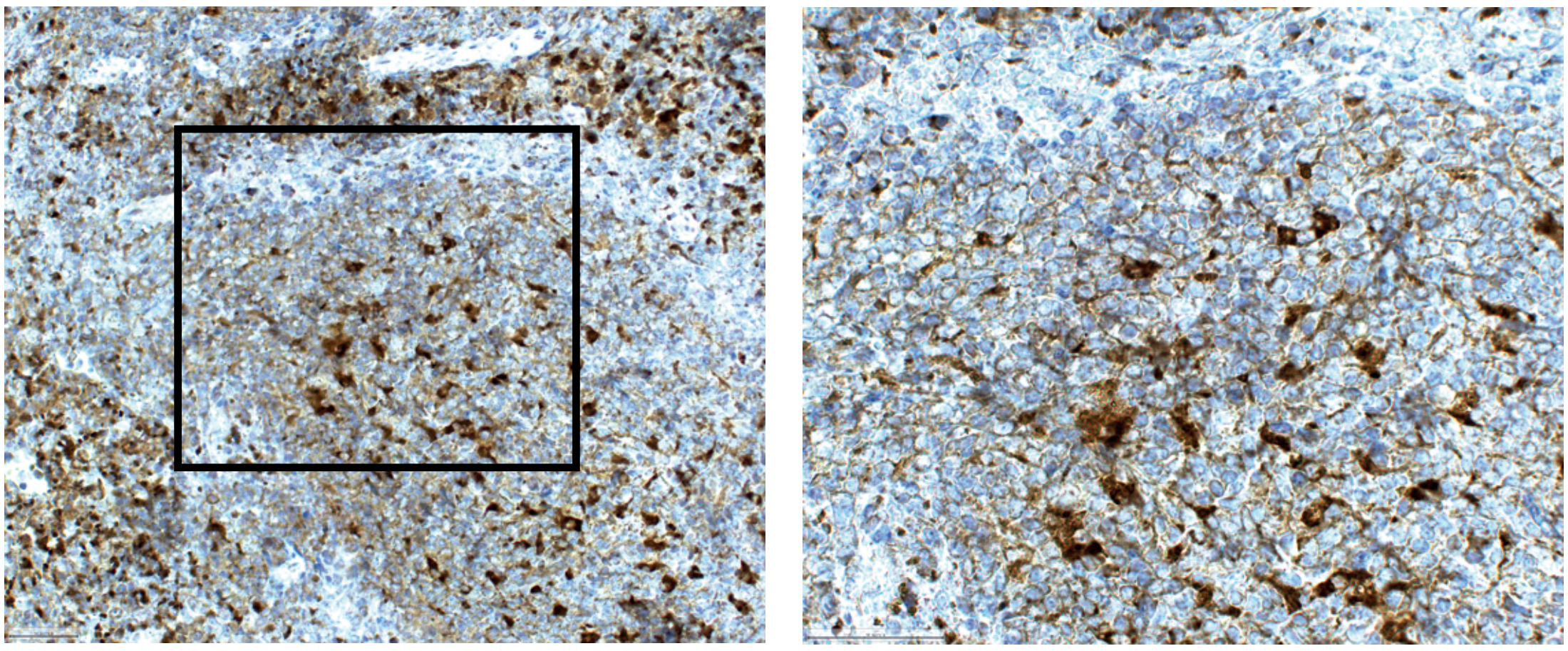

Negative Control WT Bone

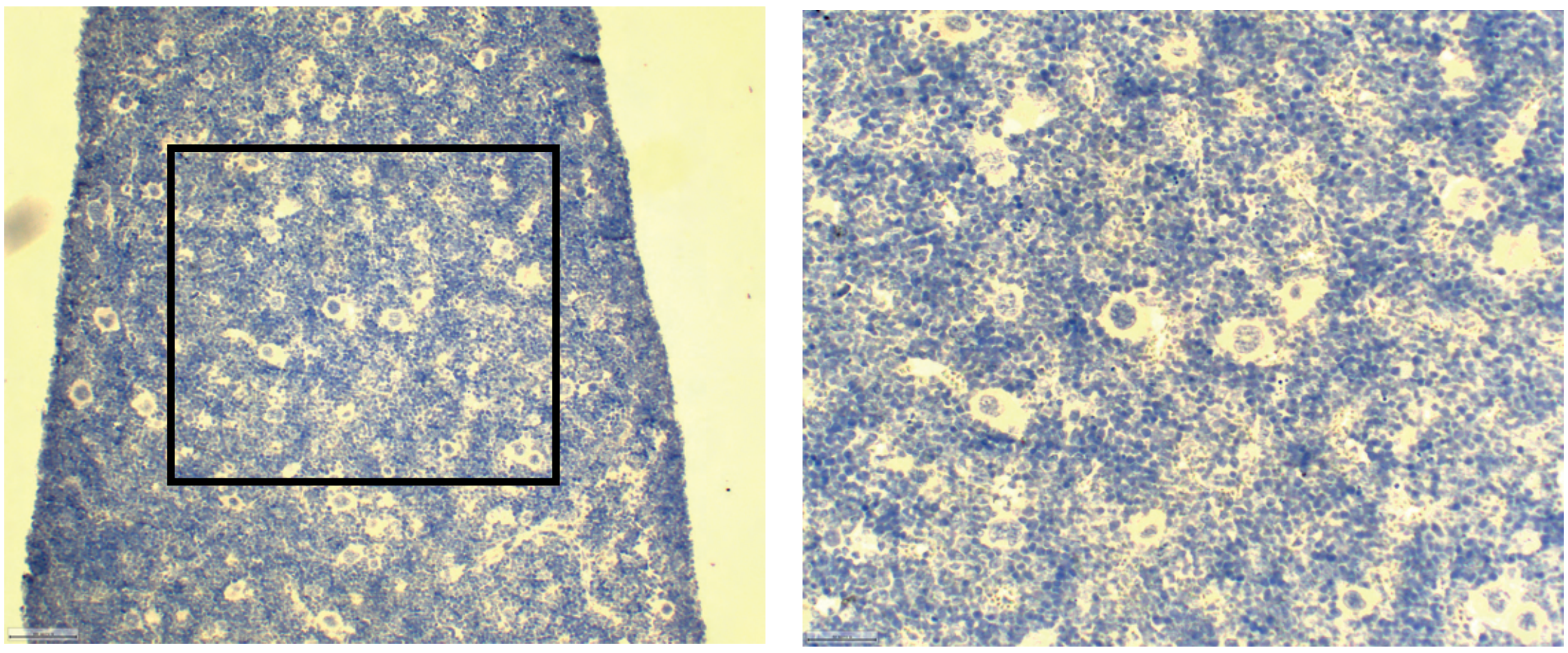

DC-SIGN (CD209) WT Bone

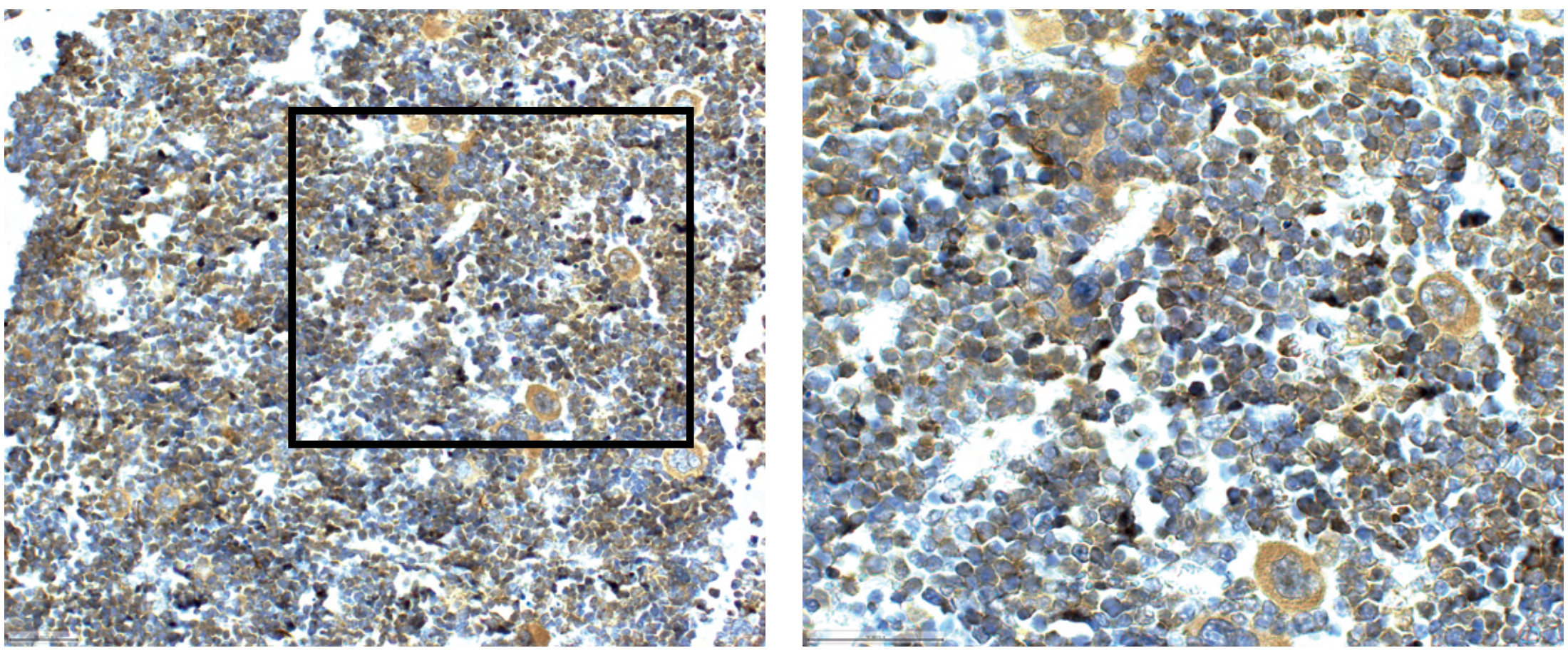

Figure S2

CEACAM1 IHC
Colon Cancer

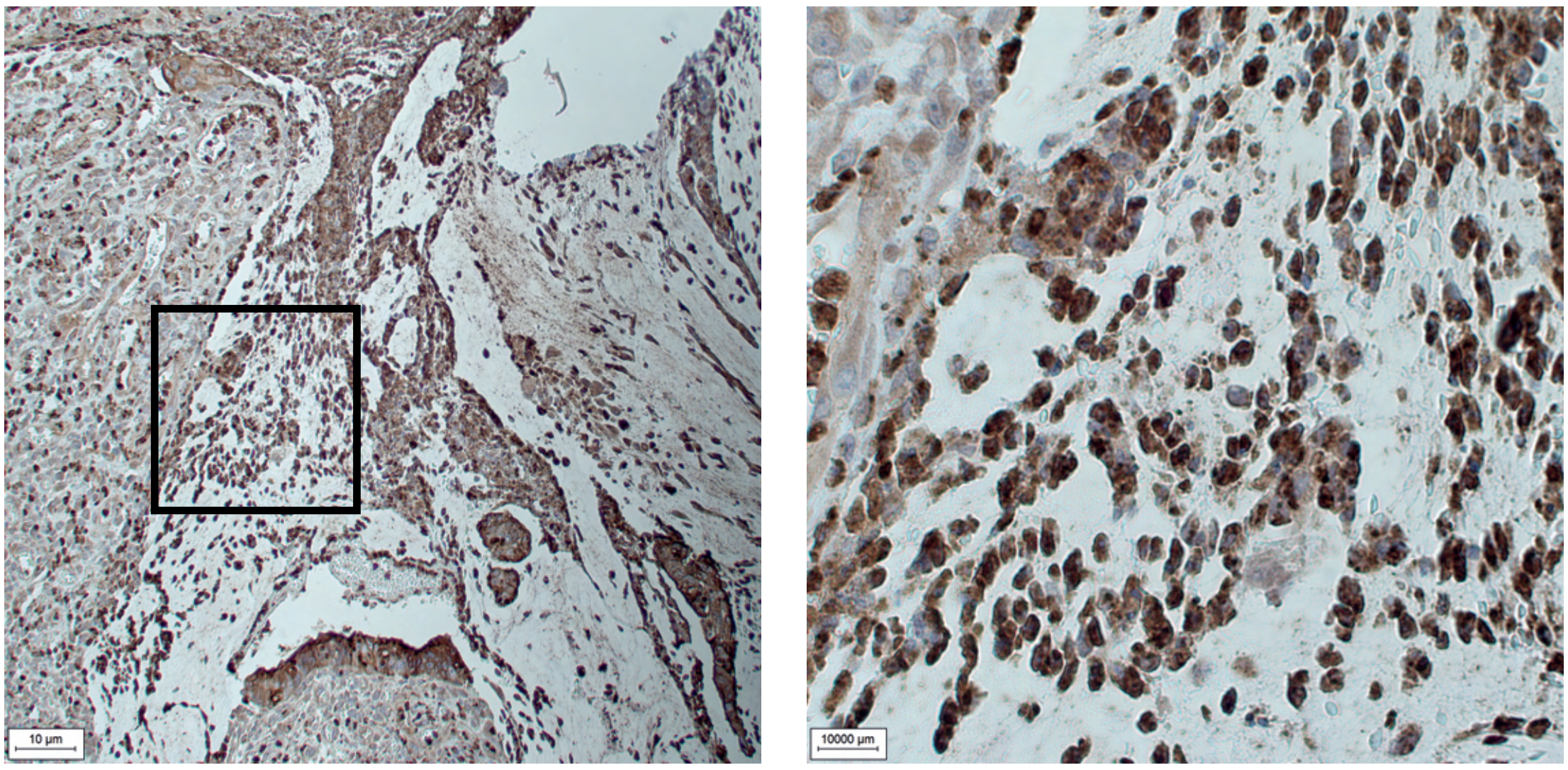

PLXNB2 IHC
Colon Cancer

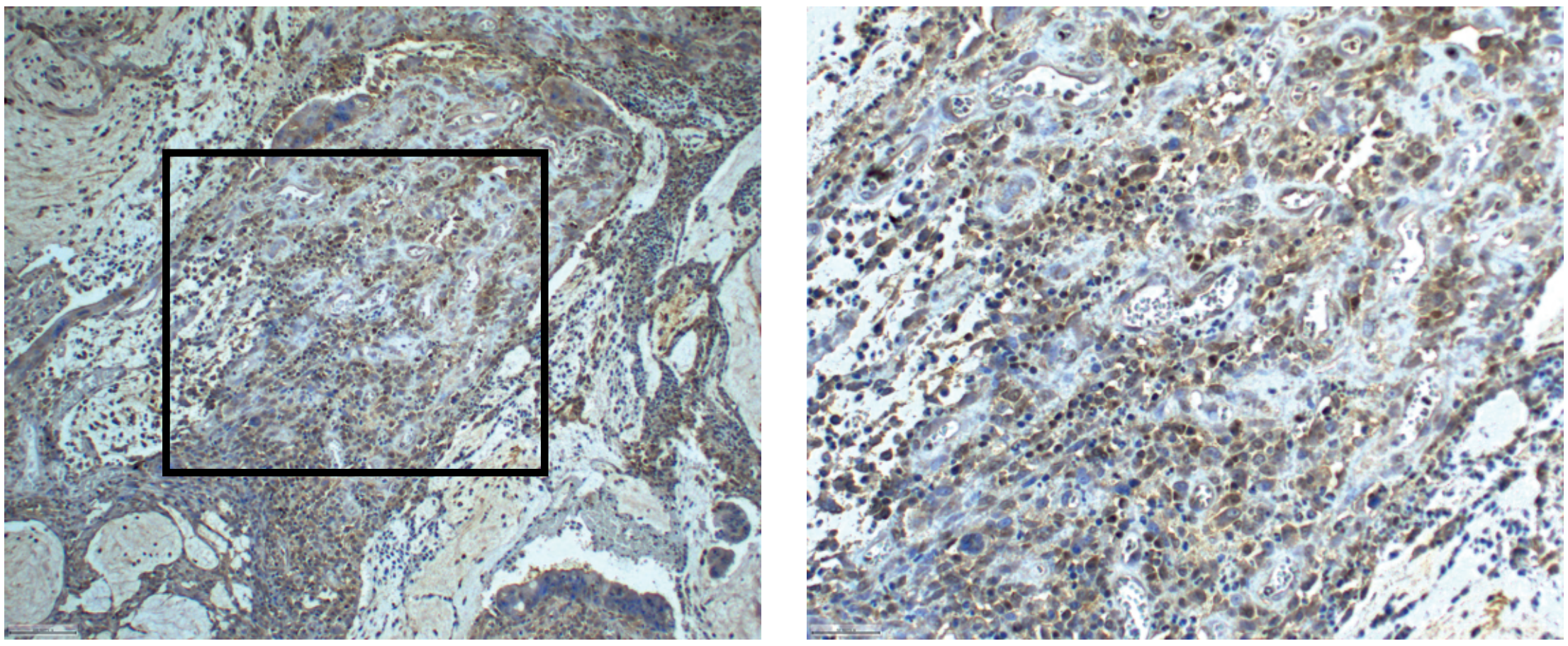

SEMA4D IHC
Lymph Nodes

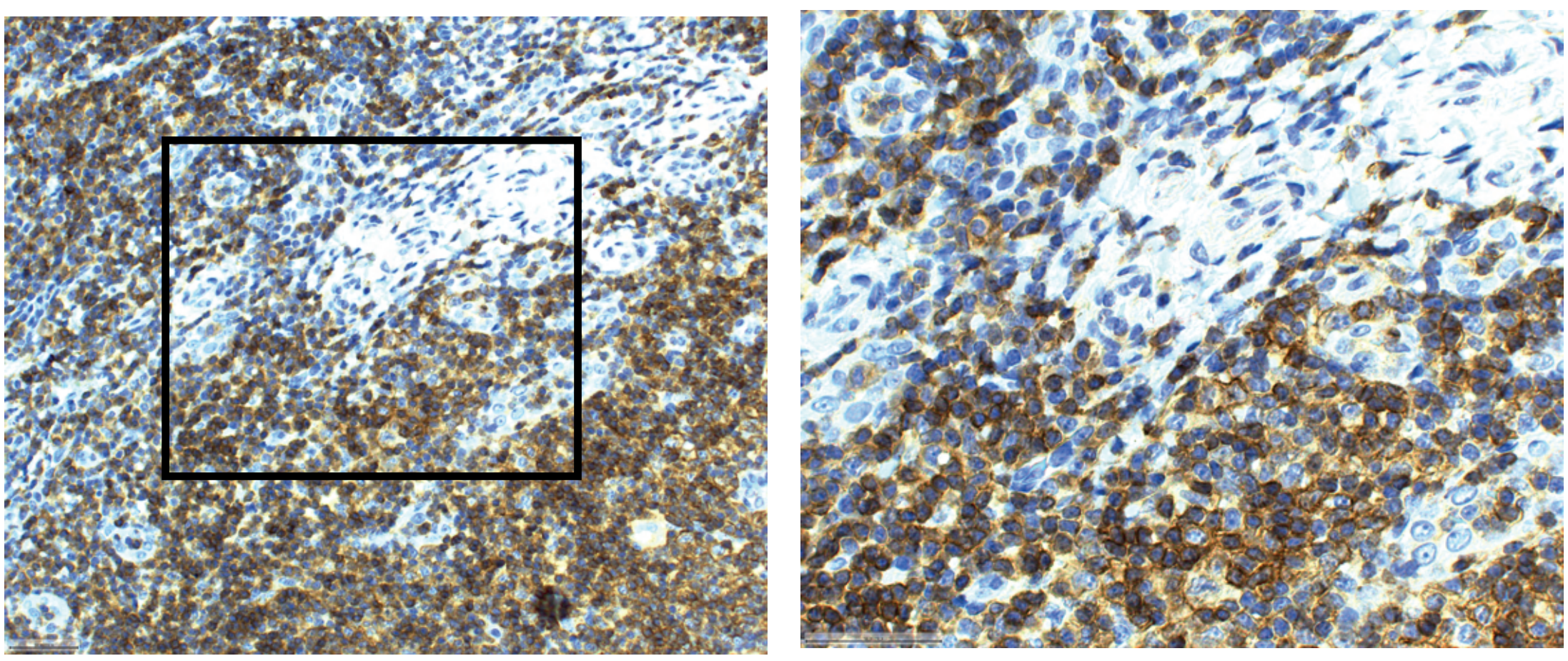

Figure S3

N-MYC IHC

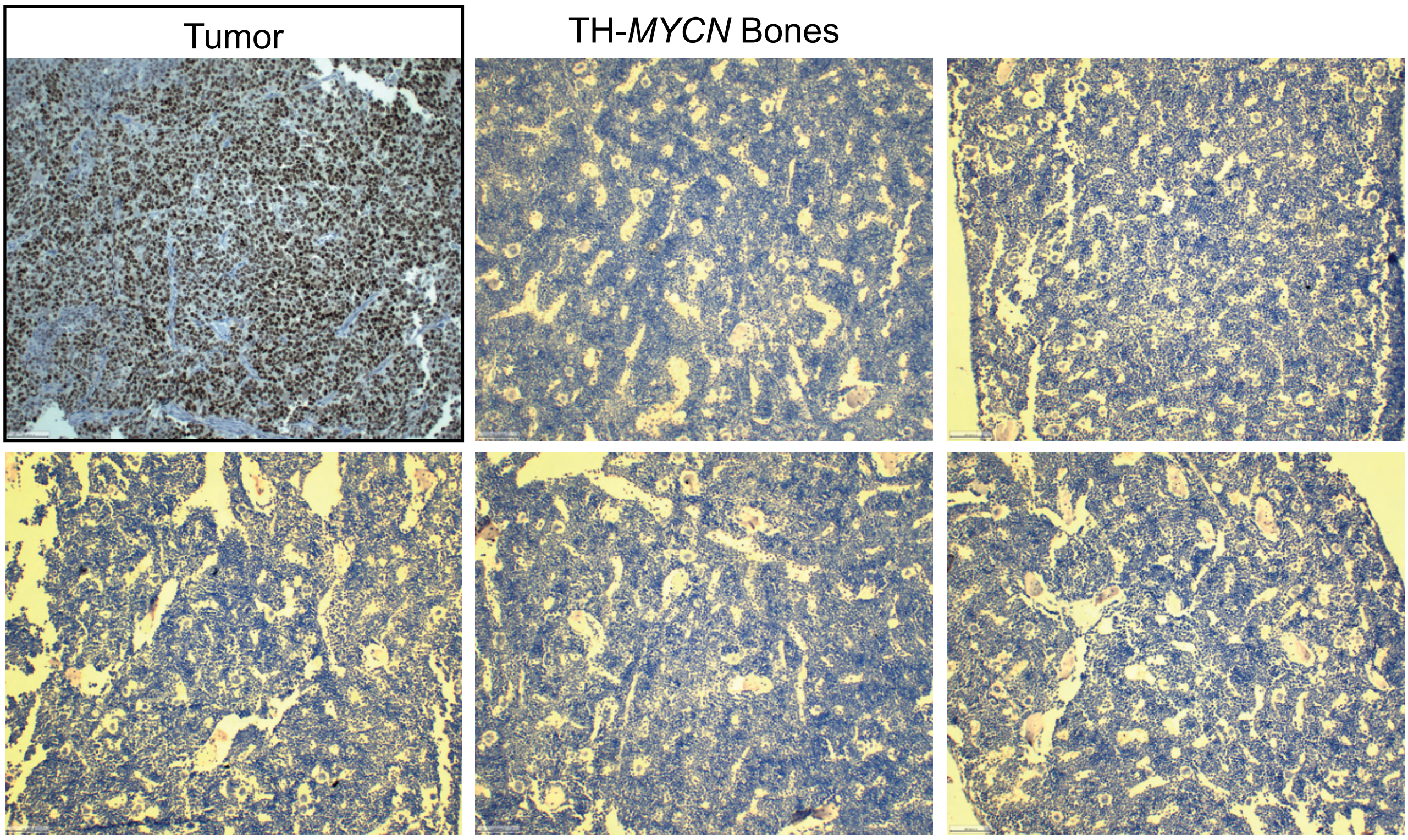

PHOX2B IHC

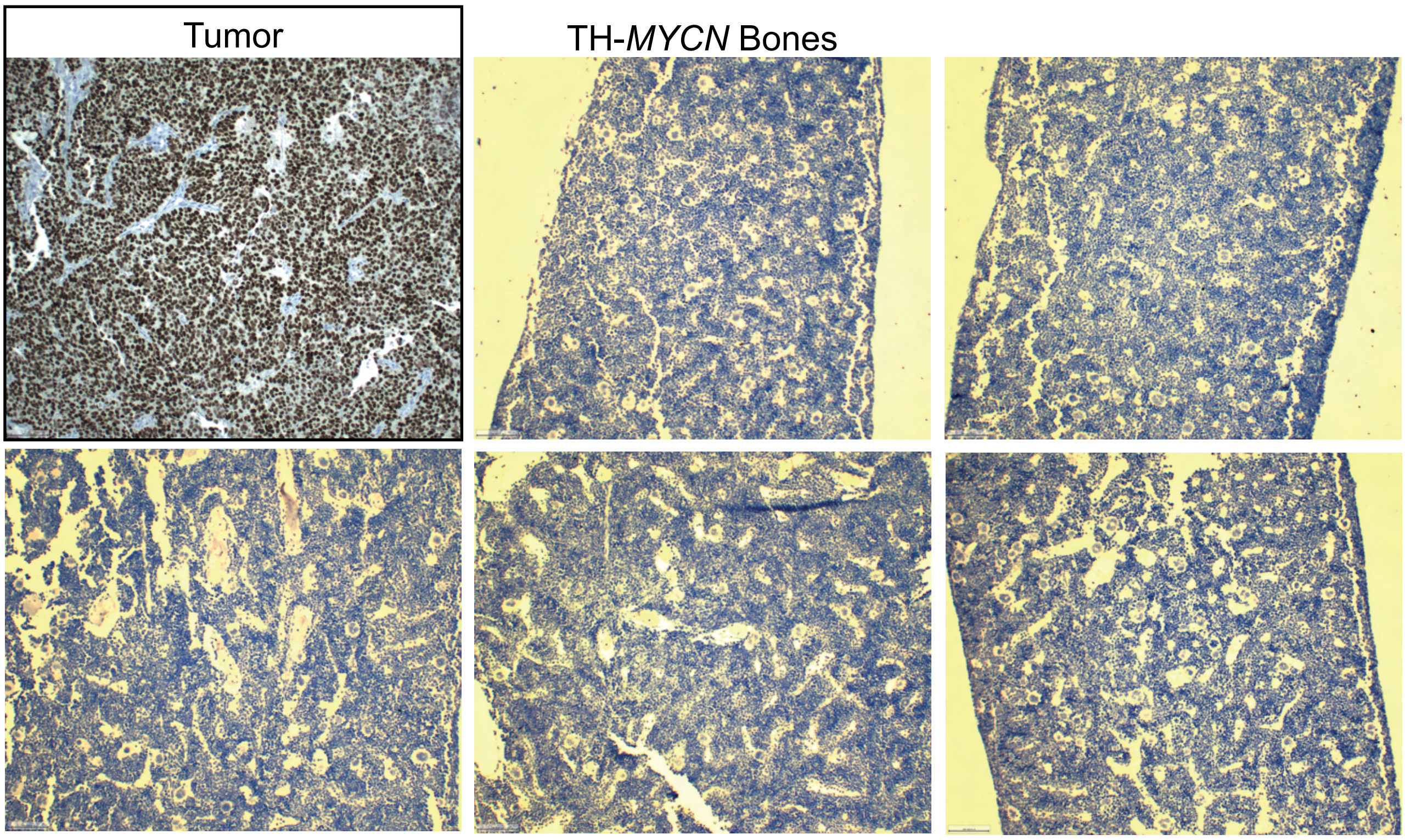

Figure S4

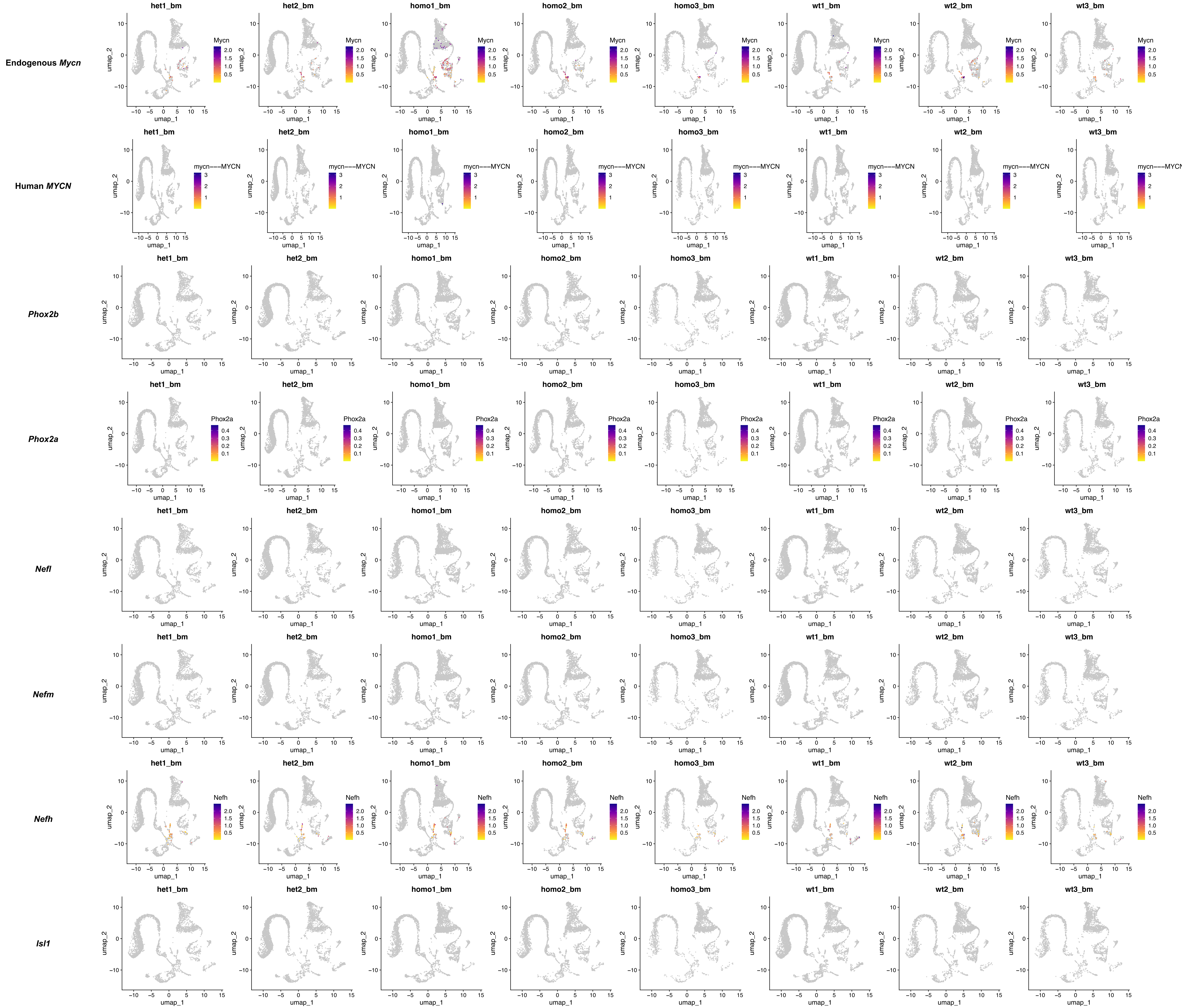
Endogenous Mycn
Human MYCN
Phox2b
Phox2a
Nefl
Nefm
Nefh
Isl1
het1_bm
het2_bm
homo1_bm
homo2_bm
homo3_bm
wt1_bm
wt2_bm
wt3_bm
umap_1
umap_2
Mycn
mycn---MYCN
Phox2a
Nefh

Figure S5

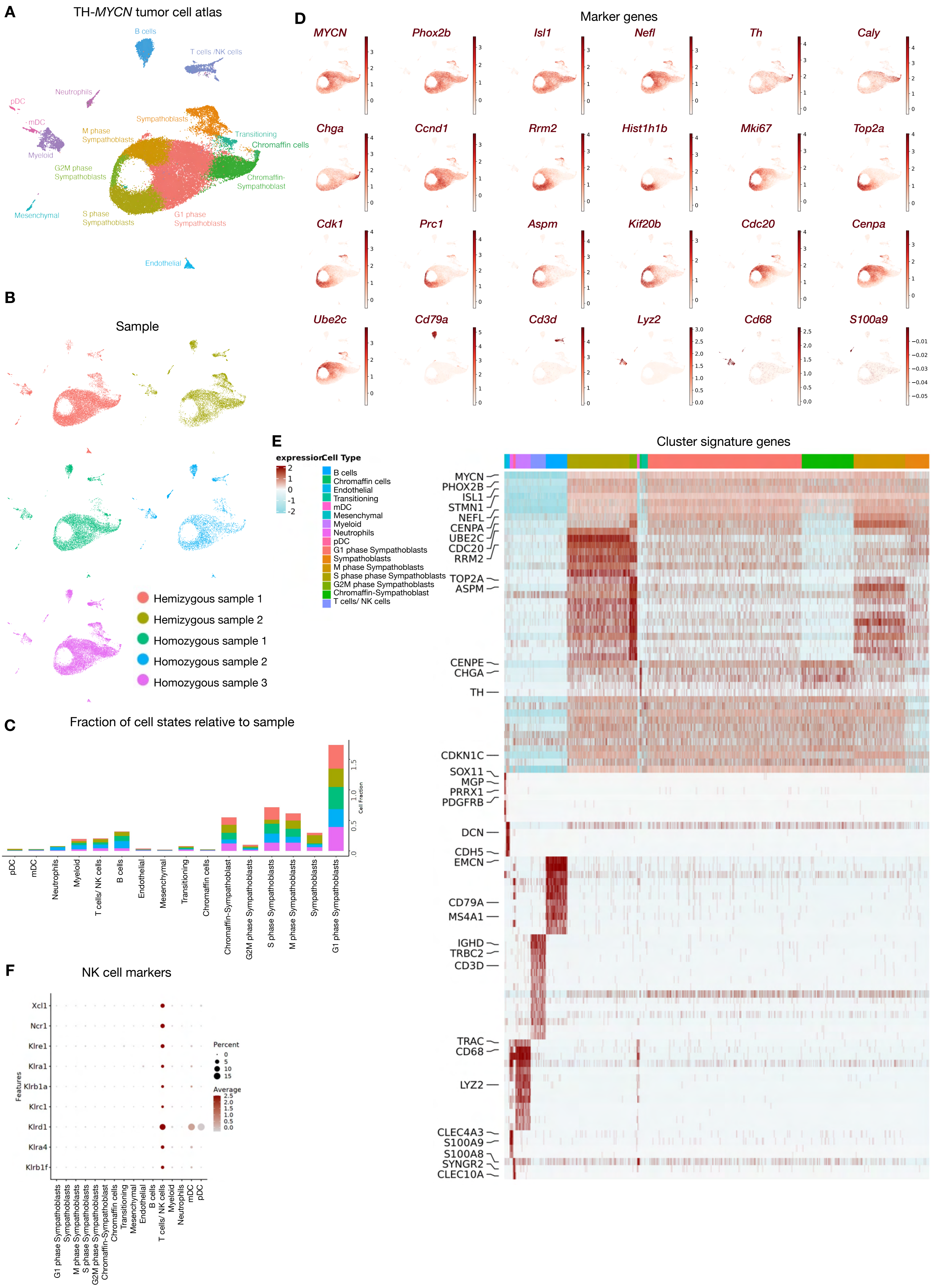

A
TH-MYCN tumor cell atlas
B cells
T cells /NK cells
Neutrophils
pDC
mDC
Myeloid
M phase Sympathoblasts
Sympathoblasts
Transitioning
Chromaffin cells
G2M phase Sympathoblasts
Chromaffin-Sympathoblast
Mesenchymal
S phase Sympathoblasts
G1 phase Sympathoblasts
Endothelial
B
Sample
Hemizygous sample 1
Hemizygous sample 2
Homozygous sample 1
Homozygous sample 2
Homozygous sample 3
C
Fraction of cell states relative to sample
Cell Fraction
pDC
mDC
Neutrophils
Myeloid
T cells/ NK cells
B cells
Endothelial
Mesenchymal
Transitioning
Chromaffin cells
Chromaffin-Sympathoblast
G2M phase Sympathoblasts
S phase Sympathoblasts
M phase Sympathoblasts
Sympathoblasts
G1 phase Sympathoblasts
D
Marker genes
MYCN
Phox2b
Isl1
Nefl
Th
Caly
Chga
Ccnd1
Rrm2
Hist1h1b
Mki67
Top2a
Cdk1
Prc1
Aspm
Kif20b
Cdc20
Cenpa
Ube2c
Cd79a
Cd3d
Lyz2
Cd68
S100a9
E
Cluster signature genes
expression
Cell Type
B cells
Chromaffin cells
Endothelial
Transitioning
mDC
Mesenchymal
Myeloid
Neutrophils
pDC
G1 phase Sympathoblasts
Sympathoblasts
M phase Sympathoblasts
S phase phase Sympathoblasts
G2M phase Sympathoblasts
Chromaffin-Sympathoblast
T cells/ NK cells
MYCN
PHOX2B
ISL1
STMN1
NEFL
CENPA
UBE2C
CDC20
RRM2
TOP2A
ASPM
CENPE
CHGA
TH
CDKN1C
SOX11
MGP
PRRX1
PDGFRB
DCN
CDH5
EMCN
CD79A
MS4A1
IGHD
TRBC2
CD3D
TRAC
CD68
LYZ2
CLEC4A3
S100A9
S100A8
SYNGR2
CLEC10A
F
NK cell markers
Features
Xcl1
Ncr1
Klre1
Klra1
Klrb1a
Klrc1
Klrd1
Klra4
Klrb1f
Percent
Average
G1 phase Sympathoblasts
Sympathoblasts
M phase Sympathoblasts
S phase Sympathoblasts
G2M phase Sympathoblasts
Chromaffin-Sympathoblast
Chromaffin cells
Transitioning
Mesenchymal
Endothelial
B cells
T cells/ NK cells
Myeloid
Neutrophils
mDC
pDC

Figure S6

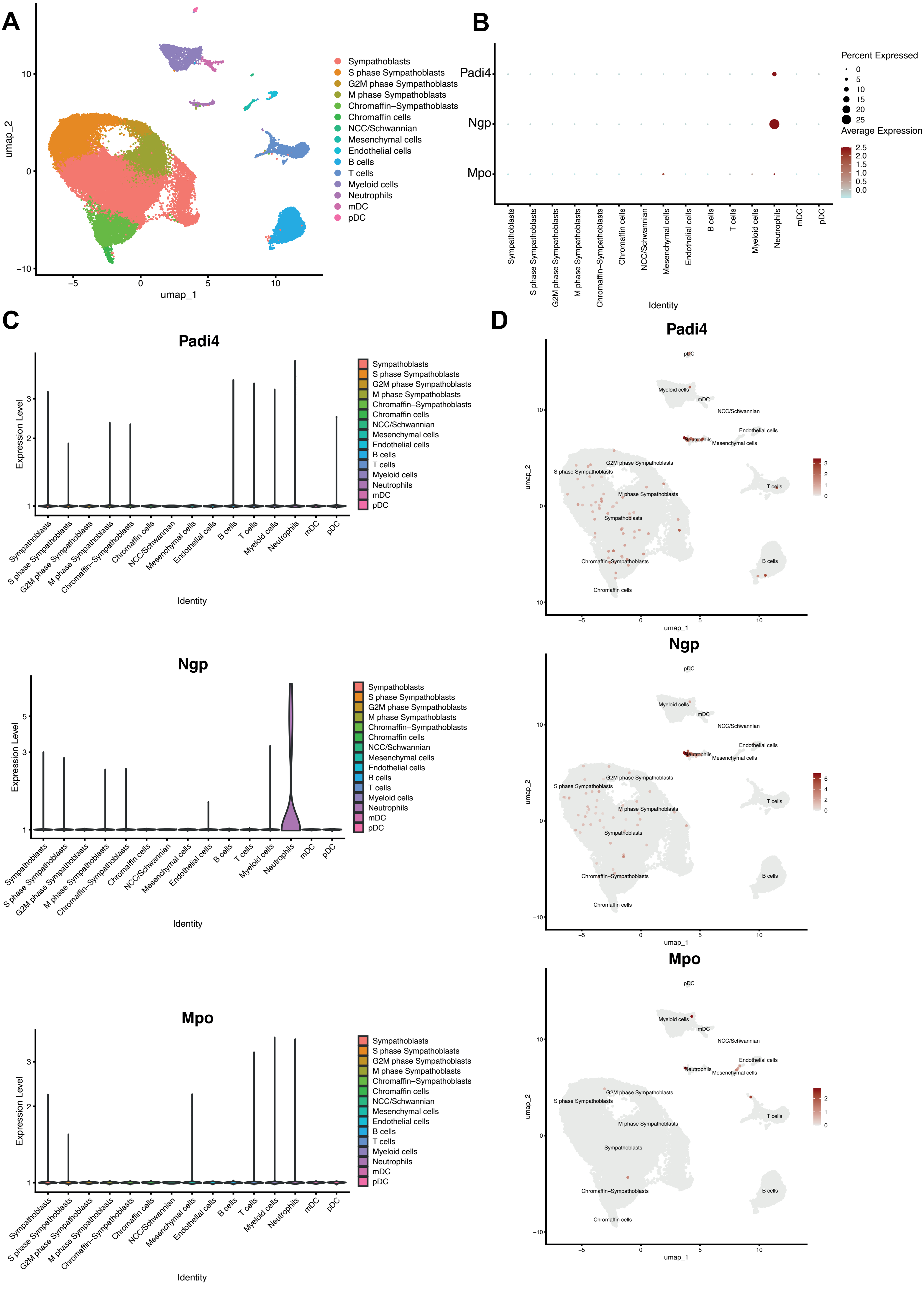
A
B
C
D
umap_1
umap_2
Sympathoblasts
S phase Sympathoblasts
G2M phase Sympathoblasts
M phase Sympathoblasts
Chromaffin–Sympathoblasts
Chromaffin cells
NCC/Schwannian
Mesenchymal cells
Endothelial cells
B cells
T cells
Myeloid cells
Neutrophils
mDC
pDC
Padi4
Ngp
Mpo
Percent Expressed
Average Expression
Identity
Expression Level

Figure S7

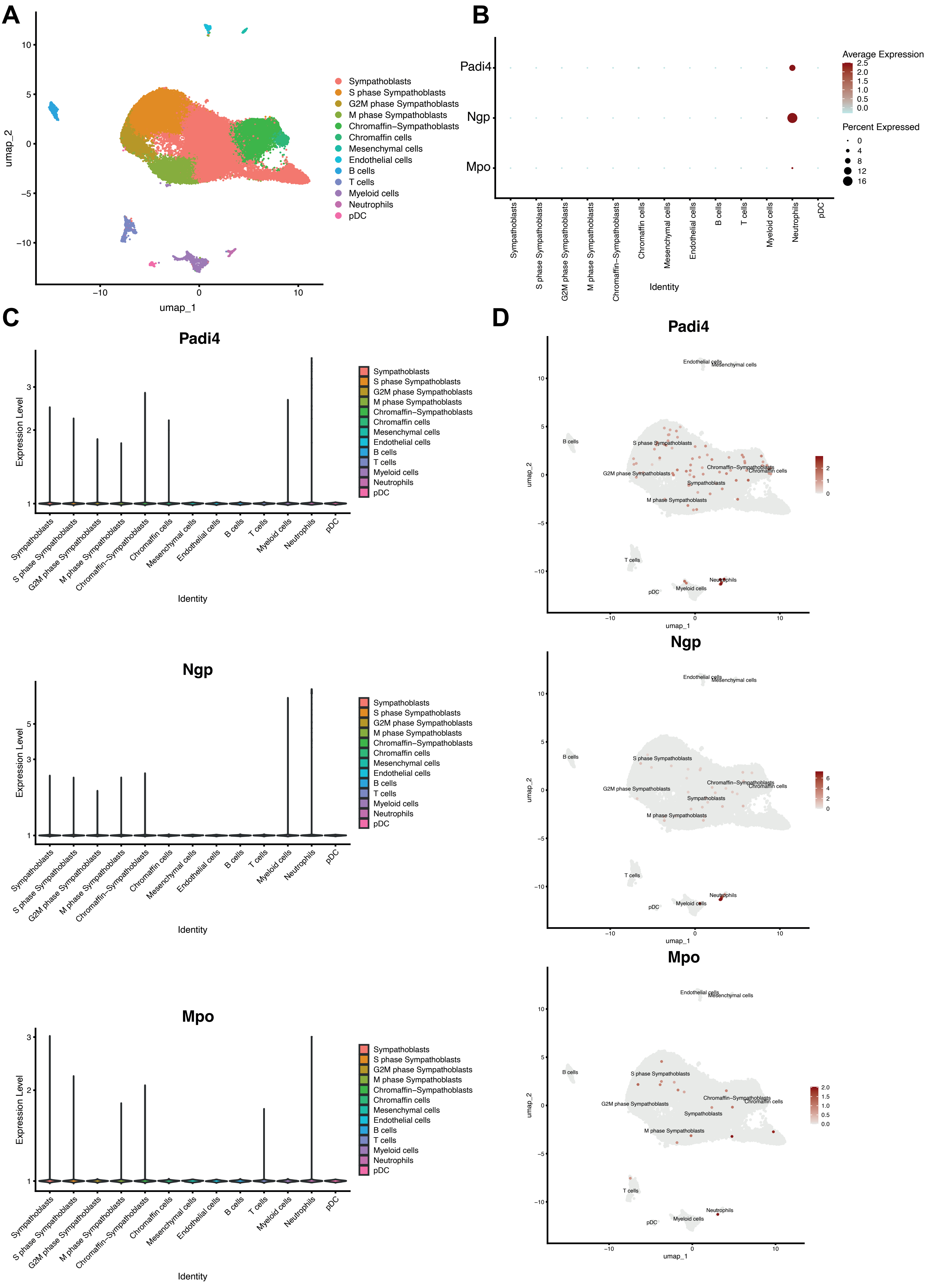

A
B
C
D
umap_1
umap_2
Sympathoblasts
S phase Sympathoblasts
G2M phase Sympathoblasts
M phase Sympathoblasts
Chromaffin–Sympathoblasts
Chromaffin cells
Mesenchymal cells
Endothelial cells
B cells
T cells
Myeloid cells
Neutrophils
pDC
Padi4
Ngp
Mpo
Identity
Average Expression
Percent Expressed
Expression Level

Figure S8

A

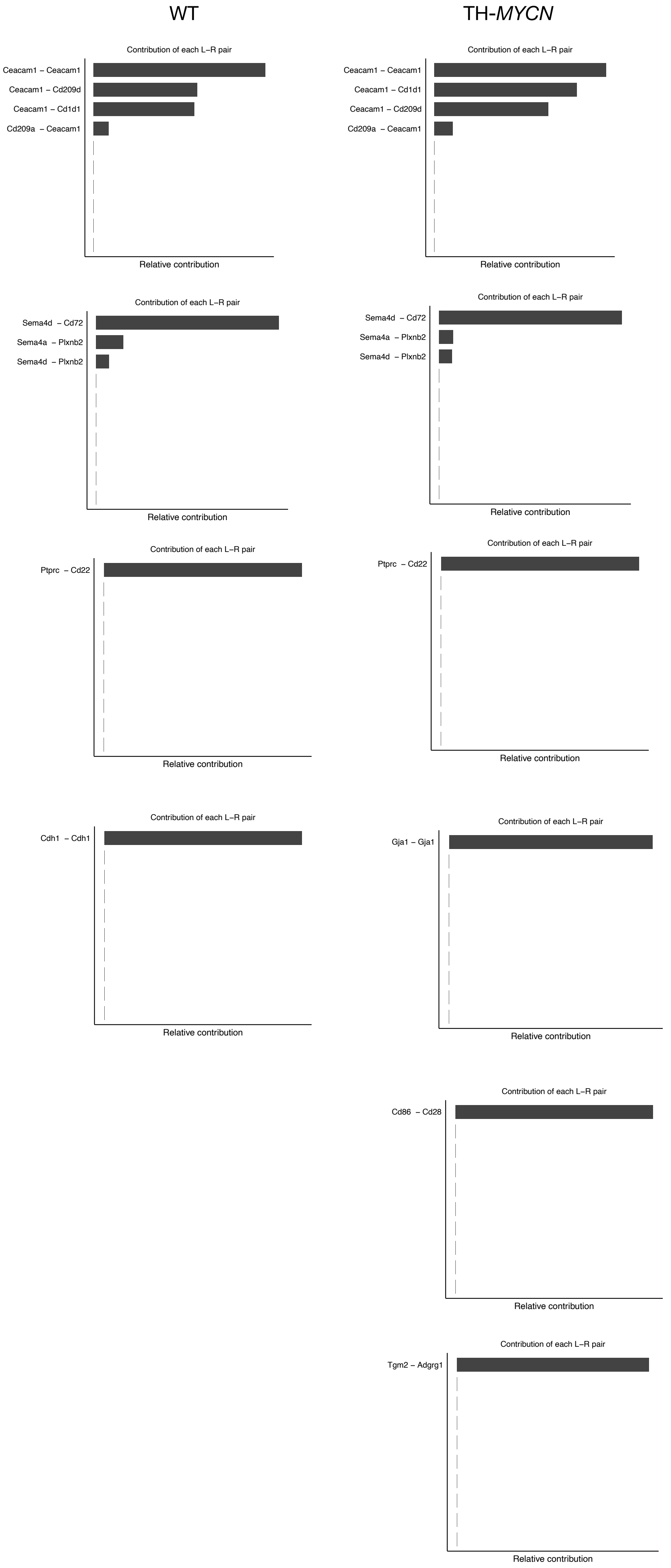

B

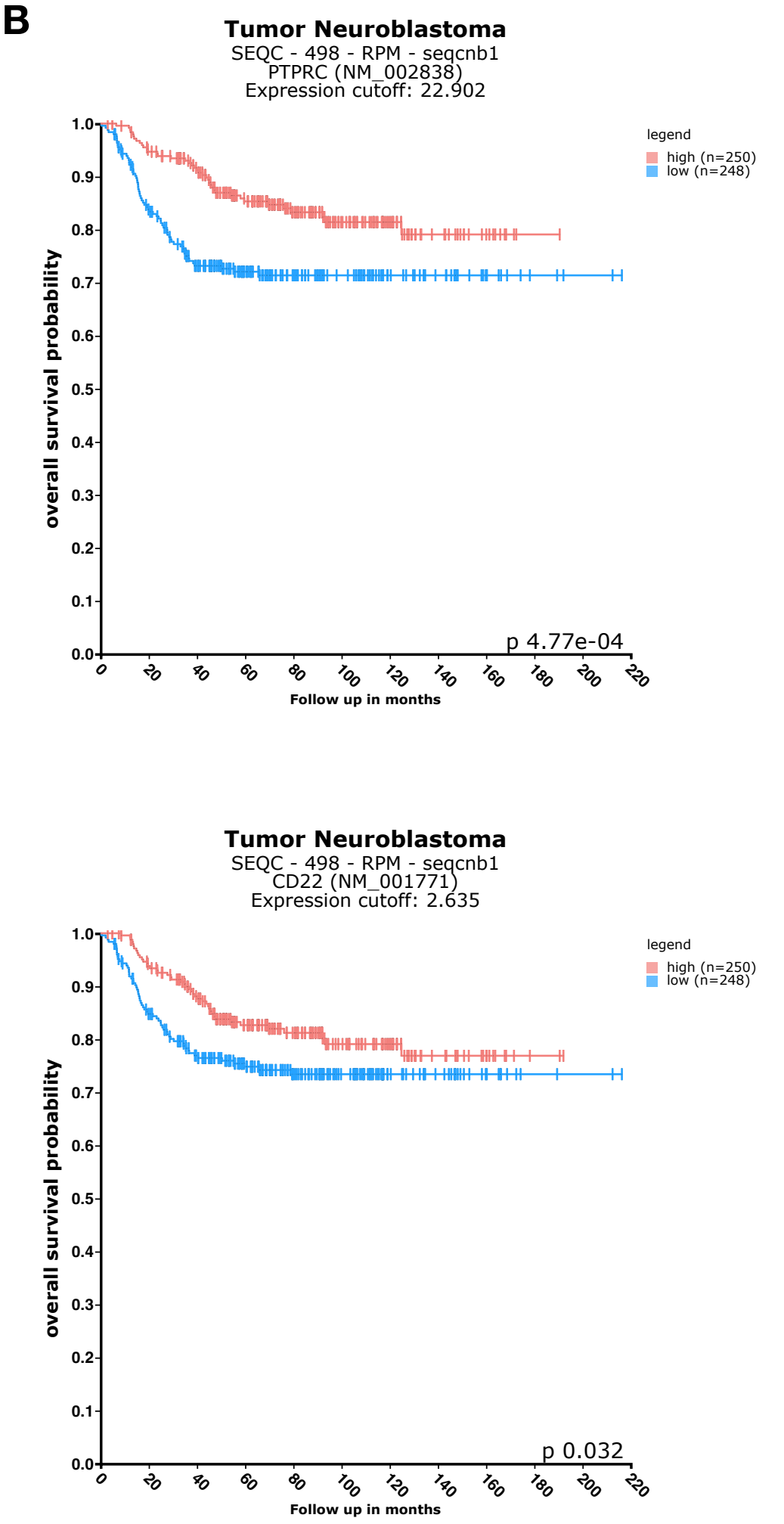

C

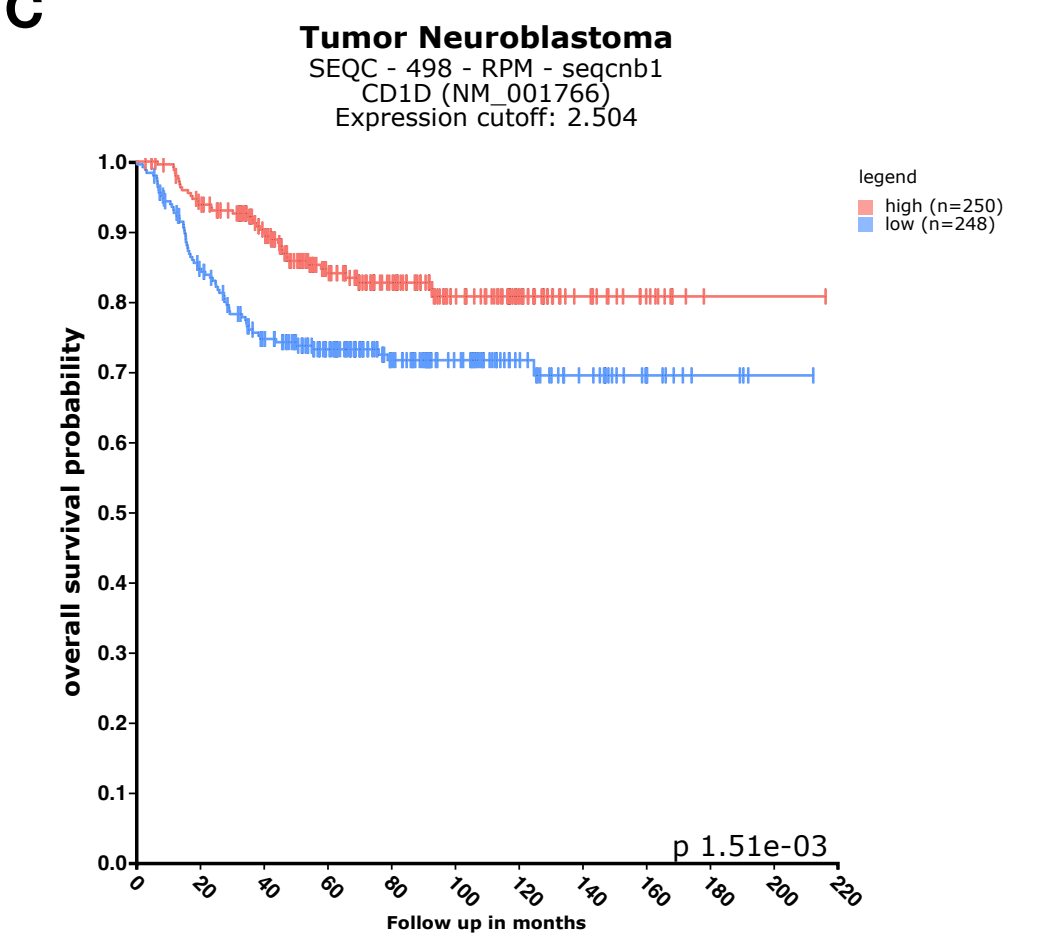

Figure S9

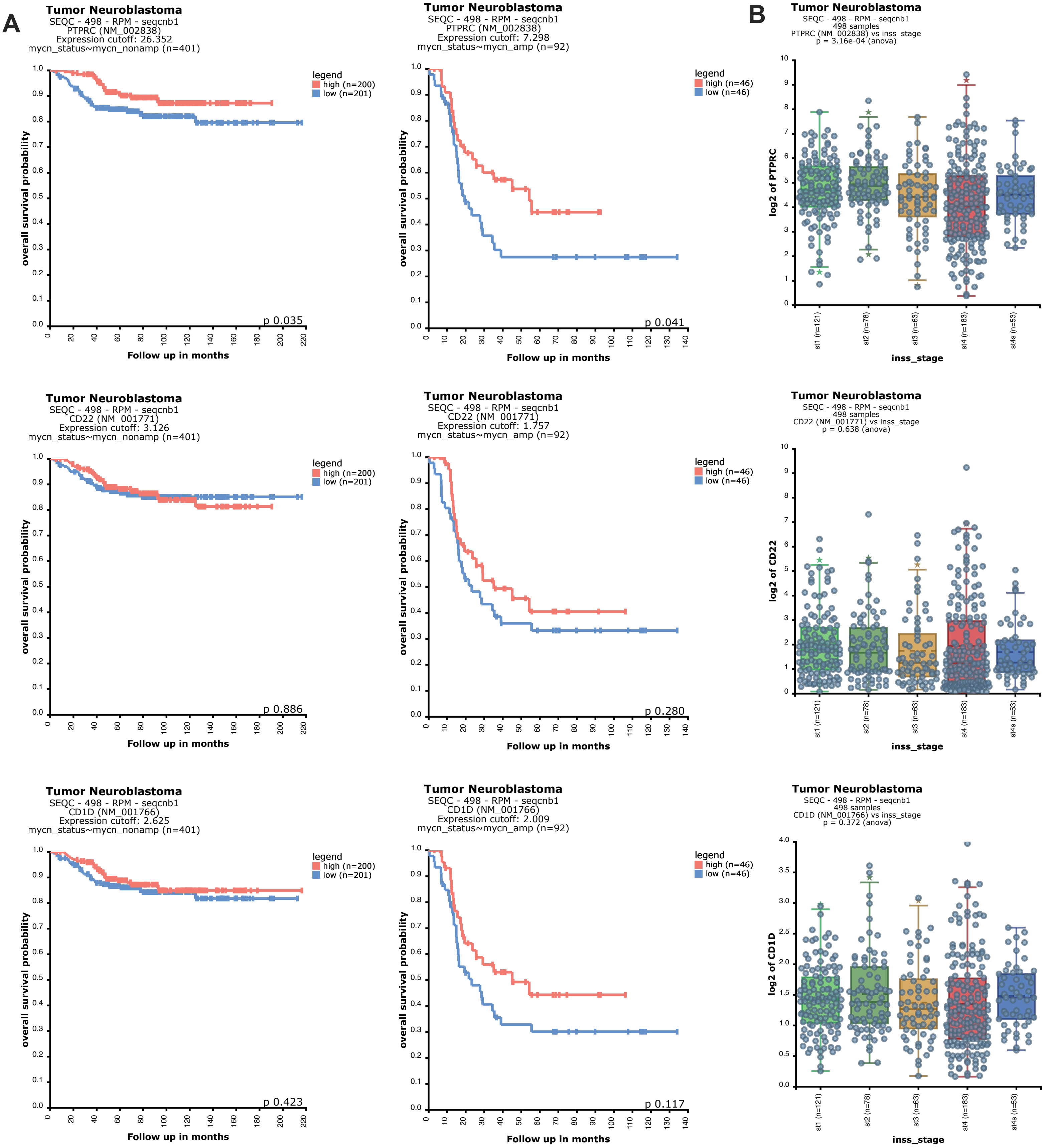
A
Tumor Neuroblastoma
SEQC - 498 - RPM - seqcnb1
PTPRC (NM_002838)
Expression cutoff: 26.352
mycn_status~mycn_nonamp (n=401)
legend
high (n=200)
low (n=201)
p 0.035
overall survival probability
Follow up in months
Tumor Neuroblastoma
SEQC - 498 - RPM - seqcnb1
PTPRC (NM_002838)
Expression cutoff: 7.298
mycn_status~mycn_amp (n=92)
legend
high (n=46)
low (n=46)
p 0.041
B
Tumor Neuroblastoma
SEQC - 498 - RPM - seqcnb1
498 samples
PTPRC (NM_002838) vs inss_stage
p = 3.16e-04 (anova)
log2 of PTPRC
st1 (n=121)
st2 (n=78)
st3 (n=63)
st4 (n=183)
st4s (n=53)
inss_stage
Tumor Neuroblastoma
SEQC - 498 - RPM - seqcnb1
CD22 (NM_001771)
Expression cutoff: 3.126
mycn_status~mycn_nonamp (n=401)
legend
high (n=200)
low (n=201)
p 0.886
Tumor Neuroblastoma
SEQC - 498 - RPM - seqcnb1
CD22 (NM_001771)
Expression cutoff: 1.757
mycn_status~mycn_amp (n=92)
legend
high (n=46)
low (n=46)
p 0.280
Tumor Neuroblastoma
SEQC - 498 - RPM - seqcnb1
498 samples
CD22 (NM_001771) vs inss_stage
p = 0.638 (anova)
log2 of CD22
Tumor Neuroblastoma
SEQC - 498 - RPM - seqcnb1
CD1D (NM_001766)
Expression cutoff: 2.625
mycn_status~mycn_nonamp (n=401)
legend
high (n=200)
low (n=201)
p 0.423
Tumor Neuroblastoma
SEQC - 498 - RPM - seqcnb1
CD1D (NM_001766)
Expression cutoff: 2.009
mycn_status~mycn_amp (n=92)
legend
high (n=46)
low (n=46)
p 0.117
Tumor Neuroblastoma
SEQC - 498 - RPM - seqcnb1
498 samples
CD1D (NM_001766) vs inss_stage
p = 0.372 (anova)
log2 of CD1D

Figure S10

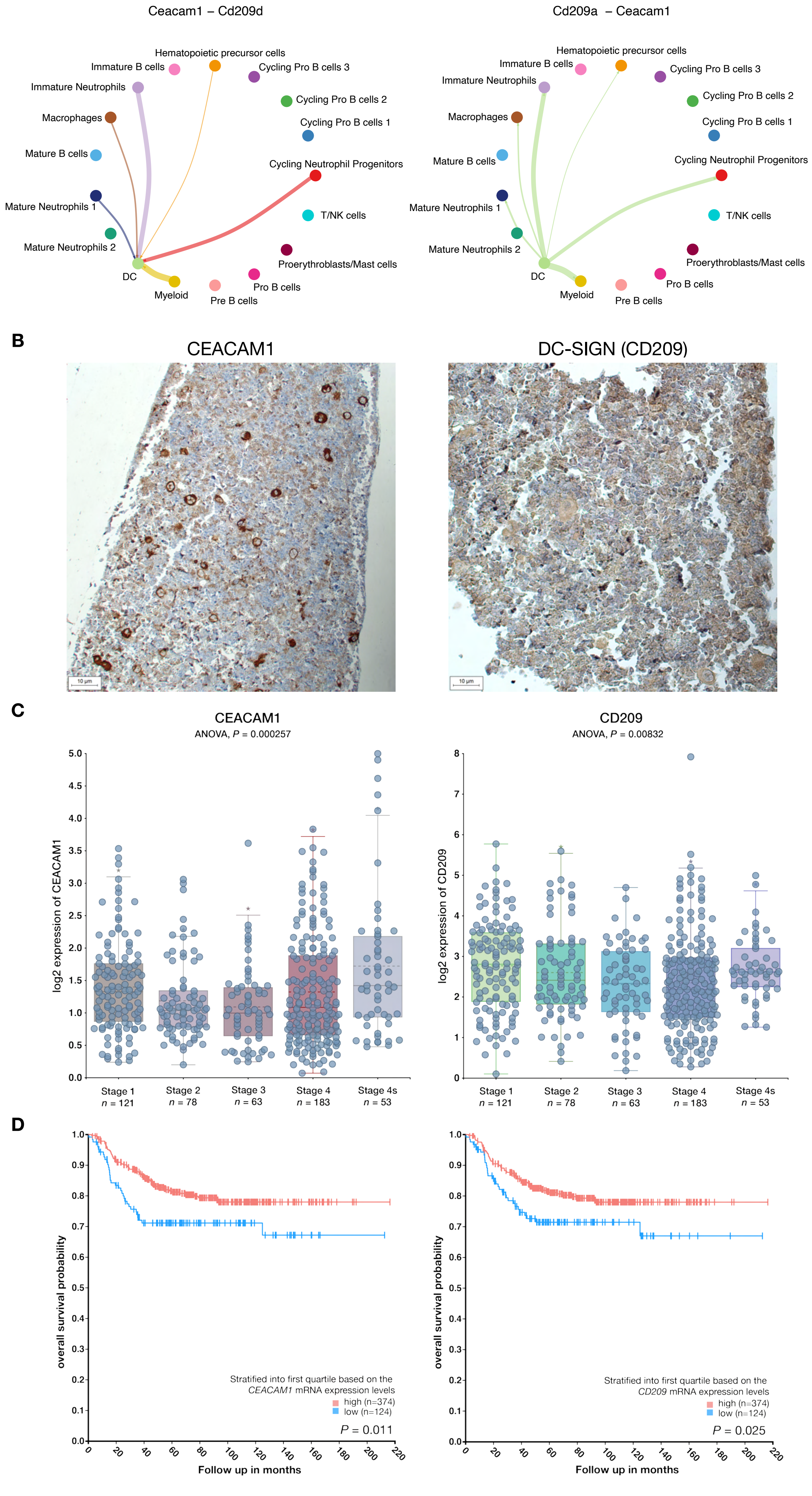

Ceacam1 – Cd209d
Cd209a – Ceacam1
Hematopoietic precursor cells
Immature B cells
Cycling Pro B cells 3
Immature Neutrophils
Cycling Pro B cells 2
Macrophages
Cycling Pro B cells 1
Mature B cells
Cycling Neutrophil Progenitors
Mature Neutrophils 1
T/NK cells
Mature Neutrophils 2
Proerythroblasts/Mast cells
DC
Pro B cells
Myeloid
Pre B cells
B
CEACAM1
DC-SIGN (CD209)
10 µm
C
CEACAM1
ANOVA, P = 0.000257
log2 expression of CEACAM1
CD209
ANOVA, P = 0.00832
log2 expression of CD209
Stage 1 n = 121
Stage 2 n = 78
Stage 3 n = 63
Stage 4 n = 183
Stage 4s n = 53
D
overall survival probability
Follow up in months
Stratified into first quartile based on the CEACAM1 mRNA expression levels
high (n=374)
low (n=124)
P = 0.011
Stratified into first quartile based on the CD209 mRNA expression levels
high (n=374)
low (n=124)
P = 0.025

Figure S11

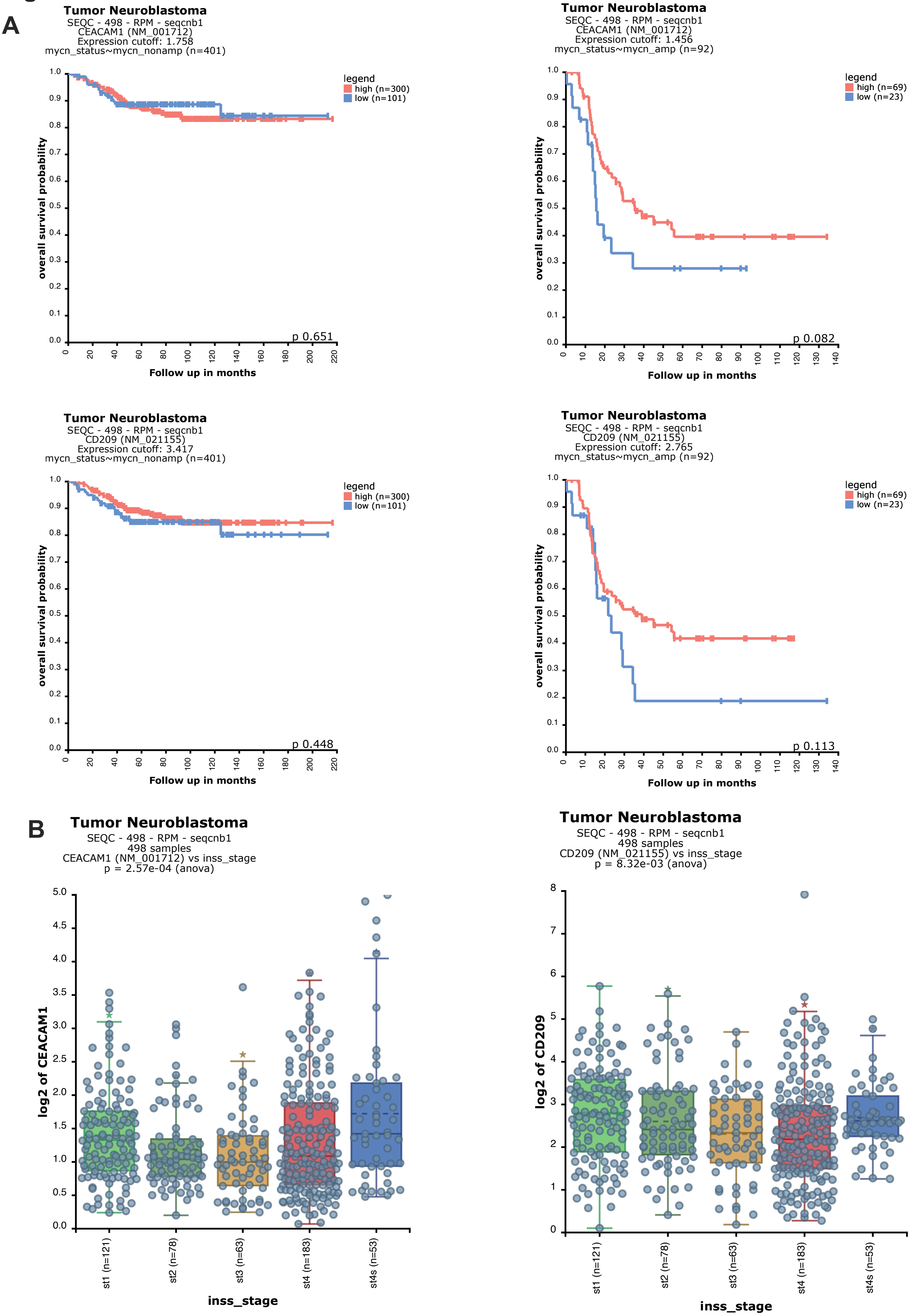

A
Tumor Neuroblastoma
SEQC - 498 - RPM - seqcnb1
CEACAM1 (NM_001712)
Expression cutoff: 1.758
mycn_status~mycn_nonamp (n=401)
legend
high (n=300)
low (n=101)
overall survival probability
Follow up in months
p 0.651
Tumor Neuroblastoma
SEQC - 498 - RPM - seqcnb1
CEACAM1 (NM_001712)
Expression cutoff: 1.456
mycn_status~mycn_amp (n=92)
legend
high (n=69)
low (n=23)
overall survival probability
Follow up in months
p 0.082
Tumor Neuroblastoma
SEQC - 498 - RPM - seqcnb1
CD209 (NM_021155)
Expression cutoff: 3.417
mycn_status~mycn_nonamp (n=401)
legend
high (n=300)
low (n=101)
overall survival probability
Follow up in months
p 0.448
Tumor Neuroblastoma
SEQC - 498 - RPM - seqcnb1
CD209 (NM_021155)
Expression cutoff: 2.765
mycn_status~mycn_amp (n=92)
legend
high (n=69)
low (n=23)
overall survival probability
Follow up in months
p 0.113
B
Tumor Neuroblastoma
SEQC - 498 - RPM - seqcnb1
498 samples
CEACAM1 (NM_001712) vs inss_stage
p = 2.57e-04 (anova)
log2 of CEACAM1
st1 (n=121)
st2 (n=78)
st3 (n=63)
st4 (n=183)
st4s (n=53)
inss_stage
Tumor Neuroblastoma
SEQC - 498 - RPM - seqcnb1
498 samples
CD209 (NM_021155) vs inss_stage
p = 8.32e-03 (anova)
log2 of CD209
st1 (n=121)
st2 (n=78)
st3 (n=63)
st4 (n=183)
st4s (n=53)
inss_stage

Figure S12

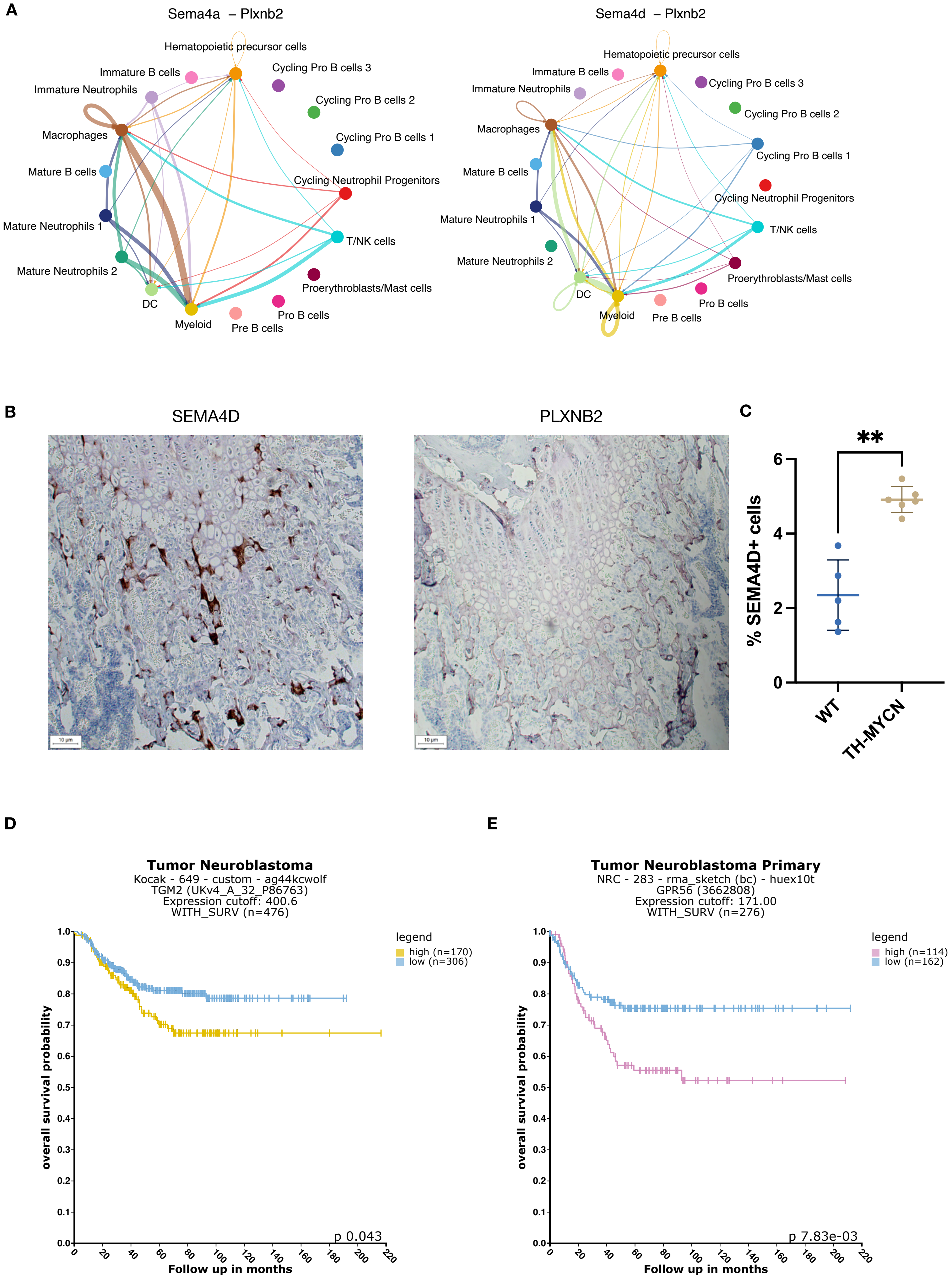

A
Sema4a – Plxnb2
Hematopoietic precursor cells
Immature B cells
Immature Neutrophils
Cycling Pro B cells 3
Cycling Pro B cells 2
Cycling Pro B cells 1
Macrophages
Mature B cells
Cycling Neutrophil Progenitors
Mature Neutrophils 1
T/NK cells
Mature Neutrophils 2
Proerythroblasts/Mast cells
DC
Myeloid
Pre B cells
Pro B cells
Sema4d – Plxnb2
Hematopoietic precursor cells
Immature B cells
Immature Neutrophils
Cycling Pro B cells 3
Cycling Pro B cells 2
Macrophages
Cycling Pro B cells 1
Mature B cells
Cycling Neutrophil Progenitors
Mature Neutrophils 1
T/NK cells
Mature Neutrophils 2
Proerythroblasts/Mast cells
DC
Myeloid
Pre B cells
Pro B cells
B
SEMA4D
PLXNB2
C
**
% SEMA4D+ cells
6
4
2
0
WT
TH-MYCN
D
Tumor Neuroblastoma
Kocak - 649 - custom - ag44kcwolf
TGM2 (UKv4_A_32_P86763)
Expression cutoff: 400.6
WITH_SURV (n=476)
legend
high (n=170)
low (n=306)
overall survival probability
p 0.043
Follow up in months
E
Tumor Neuroblastoma Primary
NRC - 283 - rma_sketch (bc) - huex10t
GPR56 (3662808)
Expression cutoff: 171.00
WITH_SURV (n=276)
legend
high (n=114)
low (n=162)
overall survival probability
p 7.83e-03
Follow up in months

Figure S13

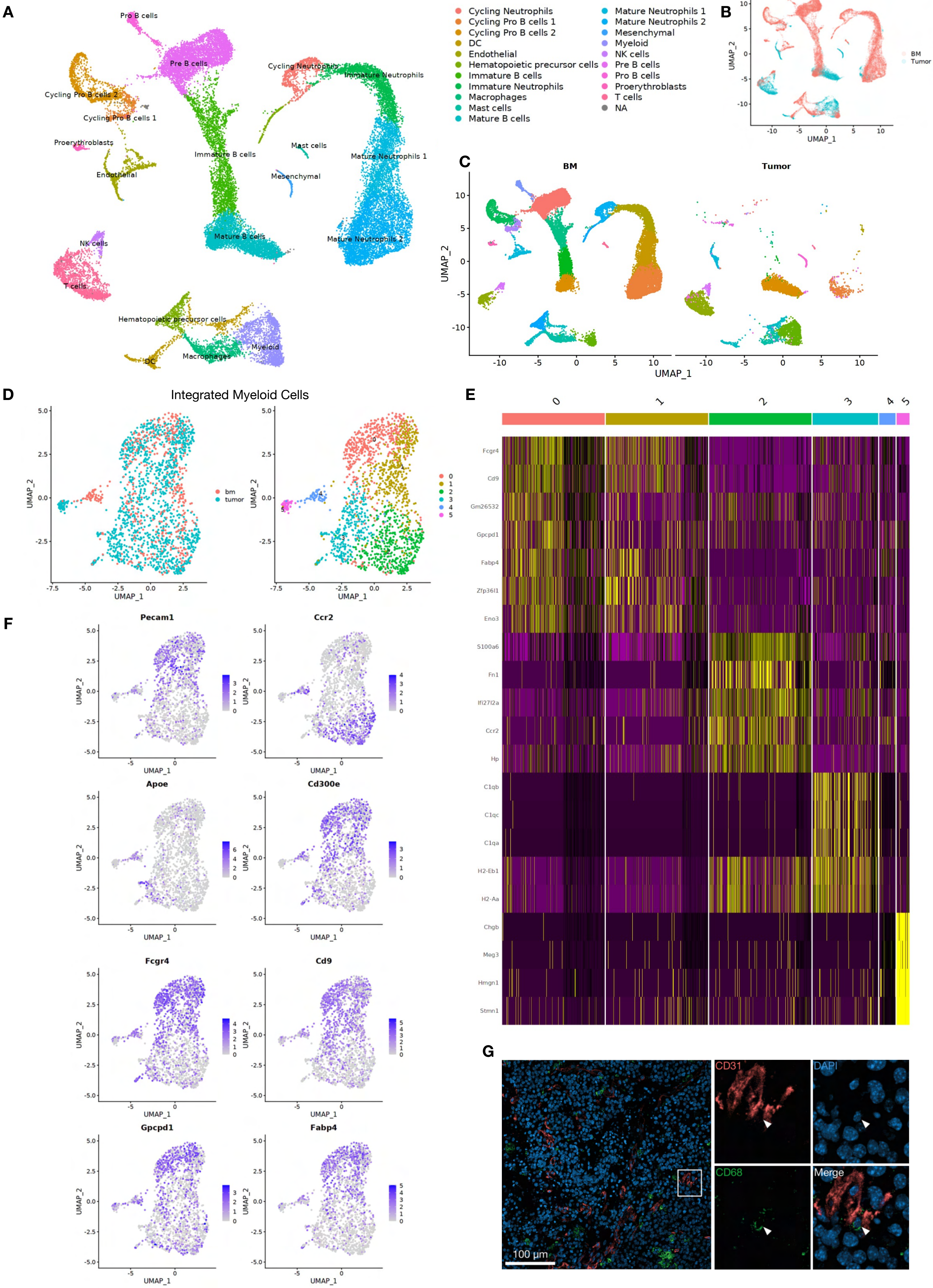

A
B
C
D
E
F
G
Cycling Neutrophils
Cycling Pro B cells 1
Cycling Pro B cells 2
DC
Endothelial
Hematopoietic precursor cells
Immature B cells
Immature Neutrophils
Macrophages
Mast cells
Mature B cells
Mature Neutrophils 1
Mature Neutrophils 2
Mesenchymal
Myeloid
NK cells
Pre B cells
Pro B cells
Proerythroblasts
T cells
NA
BM
Tumor
UMAP_1
UMAP_2
Integrated Myeloid Cells
bm
tumor
Fcgr4
Cd9
Gm26532
Gpcpd1
Fabp4
Zfp36l1
Eno3
S100a6
Fn1
Ifi27l2a
Ccr2
Hp
C1qb
C1qc
C1qa
H2-Eb1
H2-Aa
Chgb
Meg3
Hmgn1
Stmn1
Pecam1
Apoe
Cd300e
CD31
DAPI
CD68
Merge
100 μm

Figure S14

A

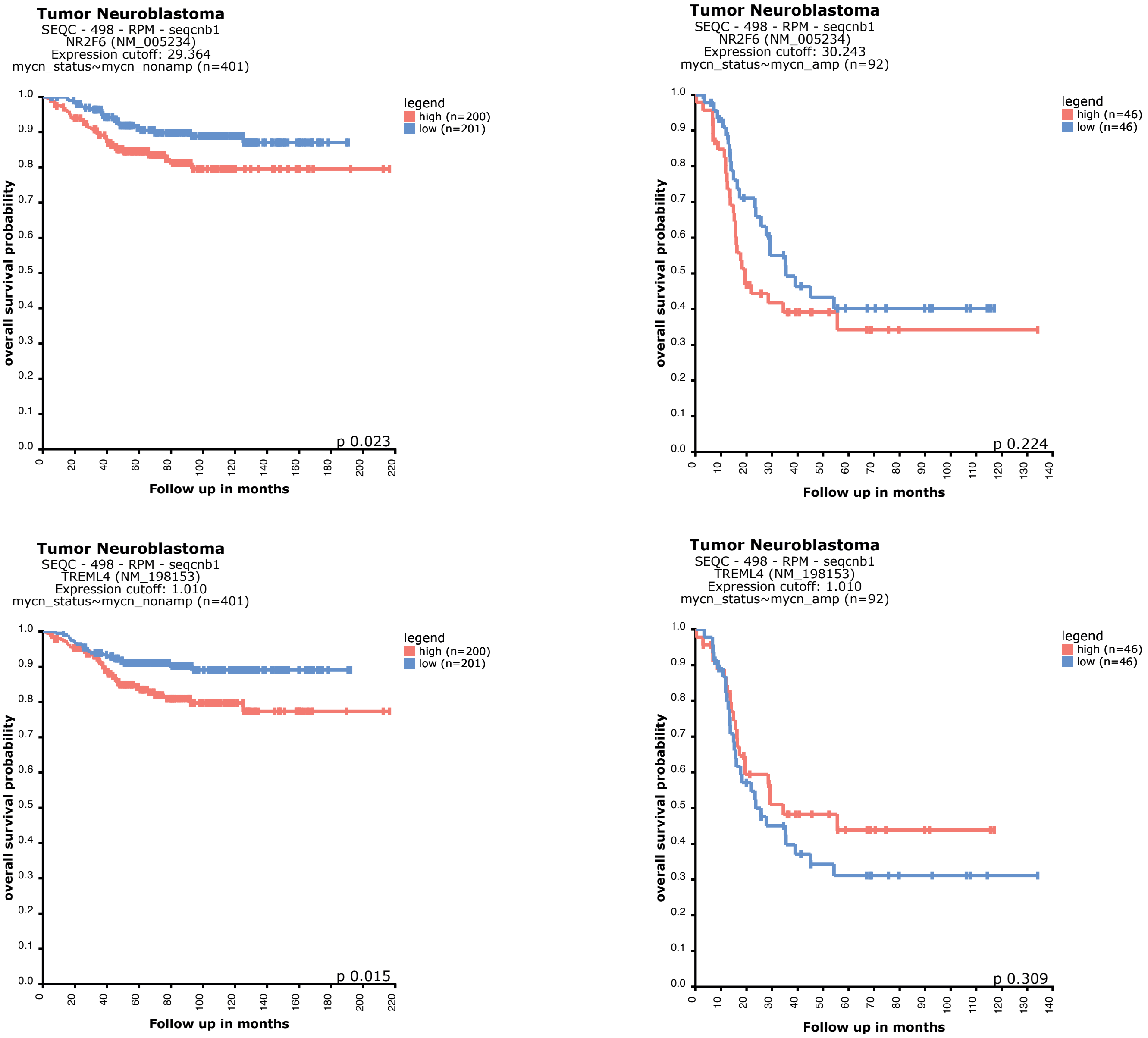

B

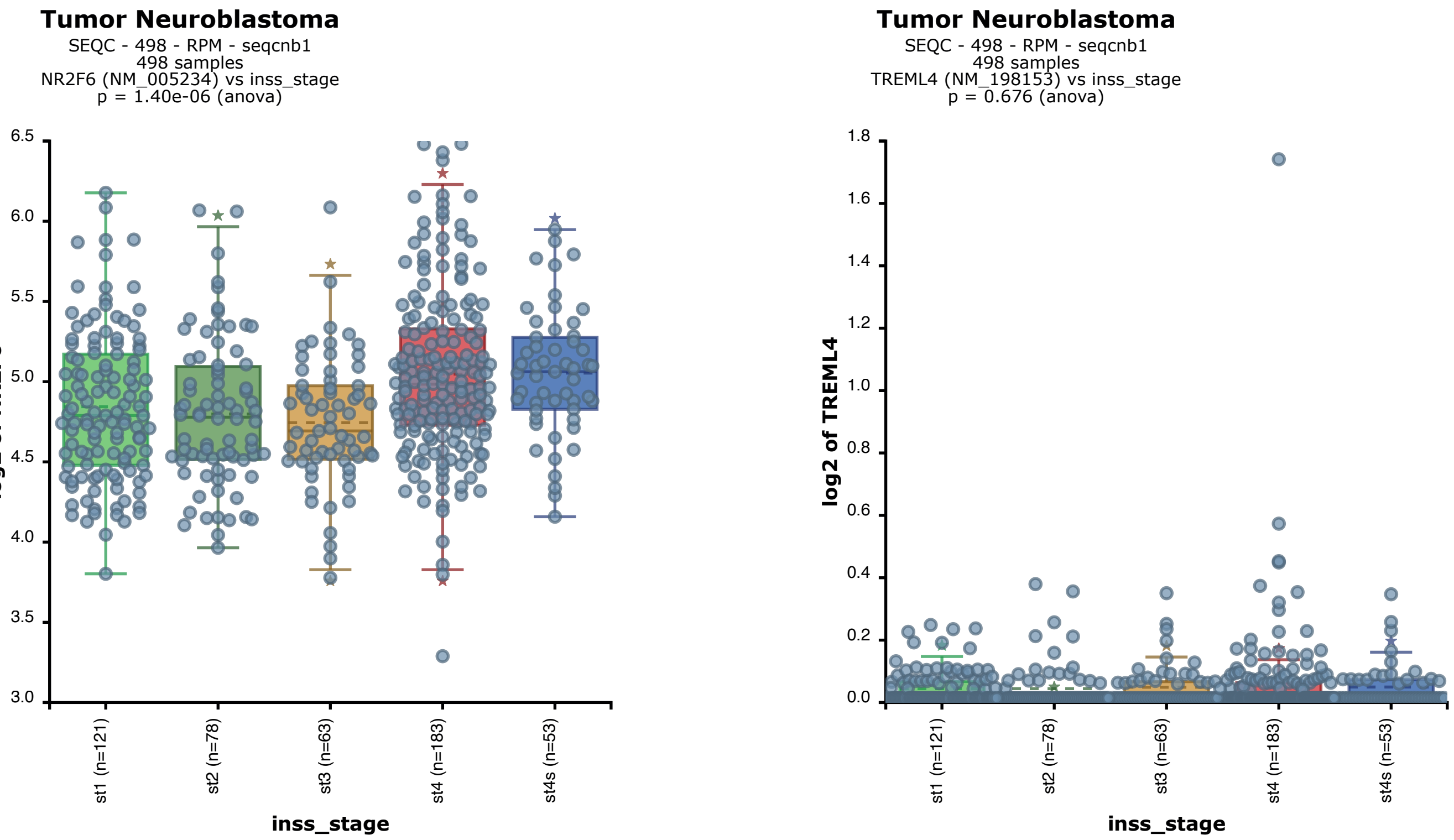

Figure S15

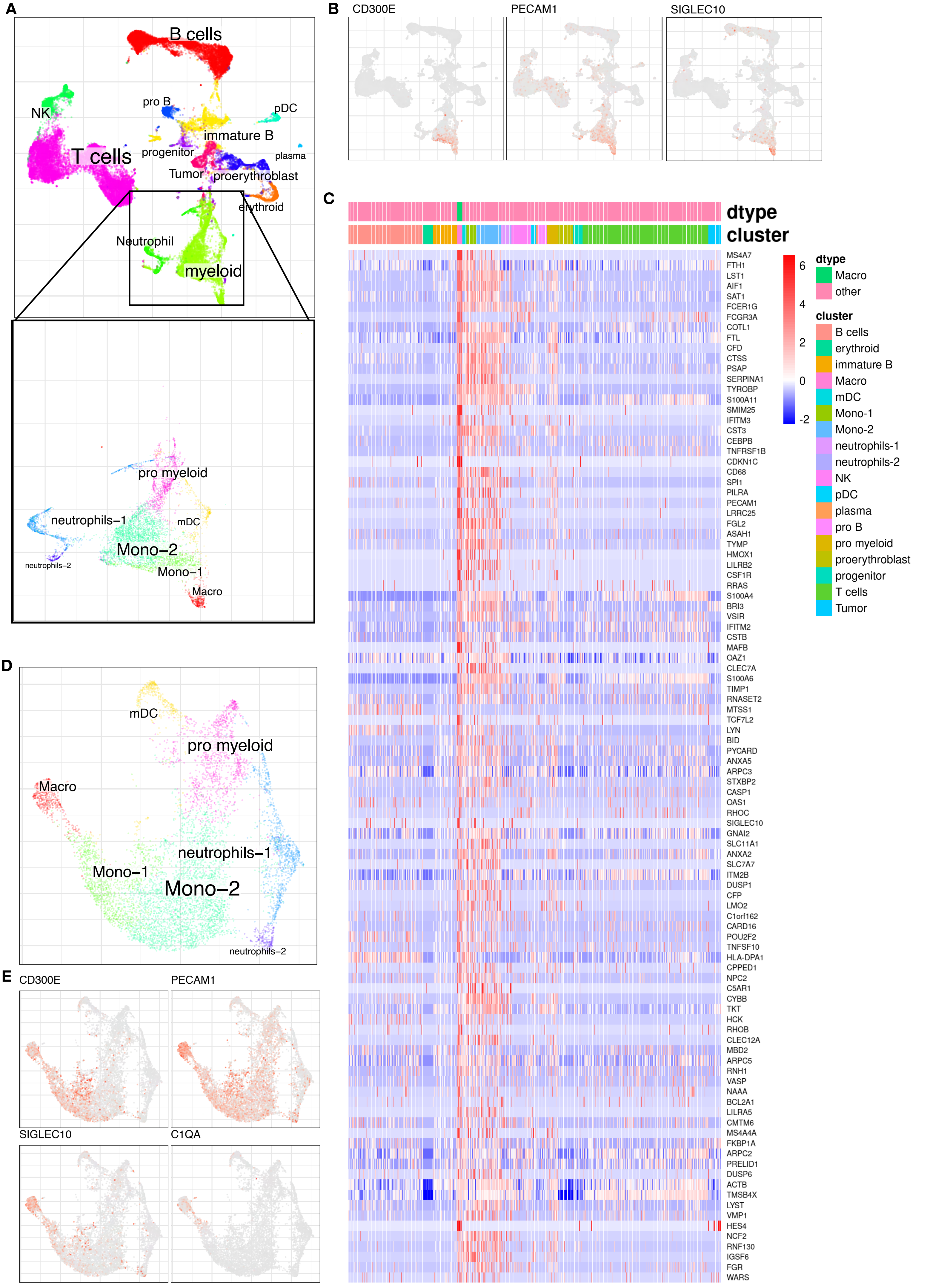

A
B cells
NK
pro B
pDC
immature B
T cells
progenitor
plasma
Tumor
proerythroblast
erythroid
Neutrophil
myeloid
pro myeloid
neutrophils−1
mDC
Mono−2
Mono−1
neutrophils−2
Macro
B
CD300E
PECAM1
SIGLEC10
C
dtype
cluster
MS4A7
FTH1
LST1
AIF1
SAT1
FCER1G
FCGR3A
COTL1
FTL
CFD
CTSS
PSAP
SERPINA1
TYROBP
S100A11
SMIM25
IFITM3
CST3
CEBPB
TNFRSF1B
CDKN1C
CD68
SPI1
PILRA
PECAM1
LRRC25
FGL2
ASAH1
TYMP
HMOX1
LILRB2
CSF1R
RRAS
S100A4
BRI3
VSIR
IFITM2
CSTB
MAFB
OAZ1
CLEC7A
S100A6
TIMP1
RNASET2
MTSS1
TCF7L2
LYN
BID
PYCARD
ANXA5
ARPC3
STXBP2
CASP1
OAS1
RHOC
SIGLEC10
GNAI2
SLC11A1
ANXA2
SLC7A7
ITM2B
DUSP1
CFP
LMO2
C1orf162
CARD16
POU2F2
TNFSF10
HLA-DPA1
CPPED1
NPC2
C5AR1
CYBB
TKT
HCK
RHOB
CLEC12A
MBD2
ARPC5
RNH1
VASP
NAAA
BCL2A1
LILRA5
CMTM6
MS4A4A
FKBP1A
ARPC2
PRELID1
DUSP6
ACTB
TMSB4X
LYST
VMP1
HES4
NCF2
RNF130
IGSF6
FGR
WARS
6
4
2
0
-2
dtype
Macro
other
cluster
B cells
erythroid
immature B
Macro
mDC
Mono-1
Mono-2
neutrophils-1
neutrophils-2
NK
pDC
plasma
pro B
pro myeloid
proerythroblast
progenitor
T cells
Tumor
D
mDC
pro myeloid
Macro
neutrophils−1
Mono−1
Mono−2
neutrophils−2
E
CD300E
PECAM1
SIGLEC10
C1QA
