# Supplementary material for: Immune remodeling and myeloid signature in the bone marrow niche of MYCN-driven neuroblastoma
Source: BJC Rep. 2026 Sep 15;4:46. doi: 10.1038/s44276-026-00249-3 (PMC13578270; doi:10.1038/s44276-026-00249-3)
Supplement: Supplementary file 2 [file 44276_2026_249_MOESM2_ESM.docx]

**SUPPLEMENTAL METHODS**

**TH-*MYCN* mouse model**

We obtained the TH-*MYCN* transgenic mice (N16 backcross to the 129X1/SvJ background) from the Mouse Models of Human Cancers Consortium Repository and kept as an inbred colony on the 129X1/SvJ background (The Jackson Laboratory). Animals were housed in standard cages at a maximum of 8 mice per cage in a temperature- and humidity-controlled room with 12-hour light/12-hour dark cycles, with ad libitum access to water and food. Monitoring of tumor development, abdominal palpations and genotyping were performed as previously described in Ref. (1). The mice were euthanized using carbon dioxide and after sacrificing the tumor, femur and tibia were resected (Table S1).

**Isolation of bone marrow and tumor cells for scRNA-seq**

Bone tissues (femur and tibia) and tumor specimens were placed in Media 199 (ThermoFisher Scientific, Ref#12350039) supplemented with 2% fetal bovine serum (FBS, ThermoFisher Scientific, Ref#10082147). Bone marrow was extracted by flushing, and mechanically fragmenting the bones for subsequent enzymatic digestion. Enzymatic dissociation of tumors and FACS enrichment of viable non-erythroid tumor and bone marrow cells was performed as described in Ref. (2).

**Single-cell library preparation and sequencing**

After sorting viable tumor and bone marrow cells, we generated single-cell libraries using the Chromium single-cell 3’ reagent kit v2 (10x Genomics), and sequenced on the Illumina NextSeq 550 platform, according to 10x Genomics protocols (<https://www.10xgenomics.com>).

**Pre-Processing and Quality Control of scRNA-Seq Data**

The raw sequencing data (FASTQ files) were aligned to a modified version of the GRCm38 mouse reference genome using Cellranger 6.1.2 (10x Genomics). To accommodate the *MYCN*-overexpressed transgenic mice, the MYCN chromosome was appended to the reference genome, creating the “*mycn__MYCN*” reference gene. Quality filtering criteria for cells included: (1) fewer than 200 total UMIs, (2) more than 7,000 UMIs, (3) over 26% mitochondrial transcript content, or (4) designation as a doublet using scDblFinder (v1.14.0).

**Data Integration and Processing of scRNA-Seq Data**

The standard Seurat pipeline was employed to integrate and process scRNA-seq datasets. This included normalization, identification of highly variable features, scaling, and dimensionality reduction. A set of 2,000 most variable genes was selected for the TH-*MYCN* bone marrow and tumor datasets. Seurat (v4.9.9) was used to mitigate batch effects and integrate the data across different samples.

**Cell Type Annotation**

FindAllMarkers function from Seurat was used to identify differentially expressed genes for each cluster. Cell-type assignment was based on the identified marker genes, cross-referenced with known markers from the literature (Table S2 and Table S3). Immune cell populations were detected in the bone marrow but no tumor cells. Heatmap of differentially expressed genes of tumor sample clusters is listed in Table S4.

**Cell-Cell Interaction Analysis**

Cell-cell interactions were inferred using CellChat (v1.5.0), leveraging the annotated single-cell dataset to analyze potential signaling pathways involved in intercellular communication.

**Patient Survival Analysis**

Survival and gene expression data from four neuroblastoma cohorts were used: SEQC-498, Versteeg-88, Kocak-649 and NRC-283. For the survival analysis, the Survival-feature (Kaplan-Meier/Cox) of the R2 database (<https://r2.amc.nl>) was applied, as described in Ref. (3).

**Fixation, decalcification and immunostaining**

Resected bones (femur and tibia) and tumors were fixed in 4% PFA overnight at 4°C. The fixed bones were decalcified in 10% EDTA solution (pH 7.8-8.0) with gentle shaking at low speed for a few days, until the bones were adequately softened. Fixed tumors and decalcified bones were then stored in 70% ethanol prior to paraffin embedding and sectioning as described in Ref. (2). Immunohistochemistry (IHC) was performed as described in Ref. (4). IHC stained sections were scanned with the ZEISS Axioscan 7 slide scanner and image analysis and quantifications were performed using QuPath version 0.5.1. GraphPad Prism version 10.4.1 for Mac (GraphPad Software, San Diego, California USA) was used for statistical analysis (non-parametric Mann–Whitney U test) and graph (Mean ± SD).

For immunofluorescence staining, sections were incubated in 60°C oven for 40 minutes, deparaffinized in xylene (2x10 minutes) and rehydrated in series of graded ethanol: 99% ethanol (2x5 minutes), 95% ethanol (1x10 minutes), 70% ethanol (1x5 minutes) and distilled water (1x10 minutes). Heat-induced epitope retrieval was performed in Target Retrieval Solution (Dako) heated at 75°C for 10 minutes, then cooled to room temperature for 20 minutes. Then, sections were washed in distilled water, followed by PBS, and finally PBS-Tween 0.01% for 5 minutes each. Sections were blocked in 2% donkey serum in PBS for 1 hour at room temperature. Primary antibodies (listed in Table S5) were diluted in PBS and incubated on sections overnight at 4°C in a dark, humid chamber. The next day, the sections were washed in PBS-Tween, followed by incubation with secondary antibodies (listed in Table S6) with DAPI diluted in PBS for 1 hour at room temperature in a dark, humid chamber. Sections were then washed in PBS-Tween and mounted using Mowiol mounting medium (Merck). Immunostaining images were acquired using a Zeiss LSM980-Airy confocal microscope with 20x, 40x (water) and 63x (oil) objectives. Images were acquired in .czi format, and ImarisViewer software v10.1.0 were used for analysis.

**References**

1.	Rasmuson A, Segerström L, Nethander M, Finnman J, Elfman LHM, Javanmardi N, et al. Tumor development, growth characteristics and spectrum of genetic aberrations in the TH-MYCN mouse model of neuroblastoma. *PloS one*. 2012;7(12): e51297. https://doi.org/10.1371/JOURNAL.PONE.0051297.

2.	Embaie BT, Sarkar H, Alchahin AM, Otte J, Olsen TK, Tümmler C, et al. Comparative Single-Cell Transcriptomics of Human Neuroblastoma and Preclinical Models Reveals Conservation of an Adrenergic Cell State. *Cancer Research*. 2025;85(6): 1015–1034. https://doi.org/10.1158/0008-5472.CAN-24-1507.

3.	Olsen TK, Dyberg C, Embaie BT, Alchahin A, Milosevic J, Ding J, et al. DHODH is an independent prognostic marker and potent therapeutic target in neuroblastoma. *JCI insight*. 2022;7(17). https://doi.org/10.1172/JCI.INSIGHT.153836.

4.	Velentza L, Wickström M, Kogner P, Ohlsson C, Zaman F, Sävendahl L. Pharmacological inhibition of BCL-2 with the FDA-approved drug venetoclax impairs longitudinal bone growth. *Scientific Reports*. 2023;13(1): 8054. https://doi.org/10.1038/S41598-023-34965-4.
