# Supplementary material for: Immune remodeling and myeloid signature in the bone marrow niche of MYCN-driven neuroblastoma
Source: BJC Rep. 2026 Sep 15;4:46. doi: 10.1038/s44276-026-00249-3 (PMC13578270; doi:10.1038/s44276-026-00249-3)
Supplement: Supplementary file 3 [file 44276_2026_249_MOESM3_ESM.docx]

**SUPPLEMENTARY**

**Supplementary Figure S1 | Immunohistochemistry staining controls**

Immunohistochemistry staining for CD209/DC-SIGN in spleen as a positive control (top), WT bone negative control without primary antibody (middle), and WT bone CD209 staining (bottom).

**Supplementary Figure S2 | Immunohistochemistry positive staining**

Immunohistochemistry positive controls for CEACAM1 and PLXNB2 in colon cancer tissue, and SEMA4D in lymph node tissue.

**Supplementary Figure S3 | Tumor marker staining**

Immunohistochemistry staining of TH-*MYCN* tumor and bone sections for N-MYC (top) and PHOX2B (bottom).

**Supplementary Figure S4 | Tumor marker gene expression**

Feature plots showing expression of neuroblastoma marker genes in individual bone marrow samples from hemizygous, homozygous, and WT mice, from left to right: hemizygous 1, hemizygous 2, homozygous 1, homozygous 2, homozygous 3, WT 1, WT 2, and WT 3. Gene names are listed in the left column: endogenous mouse *Mycn*, human *MYCN*, *Phox2b*, *Phox2a*, *Nefl*, *Nefm*, *Nefh*, and *Isl1*.

**Supplementary Figure S5 | Matched TH-*MYCN* tumor transcriptomic cell atlas.**

UMAP embedding of cells derived from five TH-*MYCN* tumors (42,332 cells). Major cell populations are colored and labelled.

(A) Distribution of cells from each sample shown on the UMAP embedding.
(B) Bar plot representing the proportion of major cell populations across samples.
(C) Feature plots of subset marker gene expression distinguishing cell types.
(D) Heatmap showing expression of top differentially expressed genes (rows) across the cells (columns) in each cluster (top color bar, as in annotated UMAP on Fig. S1A). Key genes are highlighted on the left.
(E) Dot plot of NK cell marker genes in TH-*MYCN* tumors.

**Supplementary Figure S6 | Neutrophil degranulation-related gene expression in five homozygous TH-*MYCN* tumors.**

(A) UMAP showing the major cell populations.
(B) Dot plot of *Padi4*, *Ngp*, and *Mpo*.
(C) Violin plots of *Padi4*, *Ngp*, and *Mpo* expression.
(D) Feature plots of *Padi4*, *Ngp*, and *Mpo* expression.

**Supplementary Figure S7 | Neutrophil degranulation-related gene expression in five hemizygous TH-*MYCN* tumors.**

(A) UMAP showing the major cell populations.
(B) Dot plot of *Padi4*, *Ngp*, and *Mpo*.
(C) Violin plots of *Padi4*, *Ngp*, and *Mpo* expression.
(D) Feature plots of *Padi4*, *Ngp*, and *Mpo* expression.

**Supplementary Figure S8 | Contribution of each ligand-receptor pair to the overall signaling pathways.**

(A) Bar plot of the relative contribution of each ligand-receptor pair to the CEACAM, SEMA4, CD45, CDH, GAP, CD86 and ADGRG signaling pathways in the bone marrow of WT (Left) and TH-*MYCN* mice (Right).
(B) Kaplan-Meier curve showing the overall survival in the SEQC cohort (n= 498, GEO: GSE49711), separated by median *PTPRC* or *CD22* expression.
(C) Kaplan-Meier curve showing the overall survival in the SEQC cohort (n= 498, GEO: GSE49711), separated by median *CD1D* expression.

**Supplementary Figure S9 | Survival analysis for *PTPRC*, *CD22*, and *CD1D*** **expression.**

(A) Overall survival analysis of bulk RNA-seq data from the SEQC-498 cohort (GSE49711), stratified by *MYCN* amplification status. Kaplan–Meier curves show overall survival according to *PTPRC*, *CD22*, and *CD1D* median expression.
(B) Boxplots show *PTPRC*, *CD22*, and *CD1D* expression levels stratified by INSS stage.

**Supplementary Figure S10 | CEACAM signaling in WT bone marrow.**

(A) Circle plot of the inferred Ceacam1-Cd209d (Left) and Cd209a-Ceacam1 (Right) signaling network in WT bone marrow. Arrow and edge color indicate direction. The edge thickness indicates the communication probability.
(B) Immunohistochemistry staining for CEACAM1 (Left) and DC-SIGN/CD209 (Right) in WT bone.
(C) *CEACAM1* (Left) and *CD209* (Right) expression in patients with neuroblastoma from the SEQC cohort (n= 498, GEO: GSE49711) stratified according to the INSS stages.
(D) Kaplan-Meier curve showing the overall survival in the SEQC cohort (n= 498, GEO: GSE49711), stratified into the first quartile based on the *CEACAM1*, or *CD209* mRNA expression levels.

**Supplementary Figure S11 | Survival analysis for *CEACAM1*, and *CD209*** **expression.**

(A) Overall survival analysis of bulk RNA-seq data from the SEQC-498 cohort (GSE49711), stratified by *MYCN* amplification status. Kaplan–Meier curves show overall survival stratified into the first quartile based on the CEACAM1, or CD209 mRNA expression levels.
(B) Boxplots show *CEACAM1*, and *CD209* expression levels stratified by INSS stage.

**Supplementary Figure S12 | SEMA4 signaling in TH-*MYCN* bone marrow.**

(A) Circle plot of the inferred Sema4a-Plxnb2 (Left) and Sema4d-Plxnb2 (Right) signaling network in TH-*MYCN* bone marrow. Arrow and edge color indicate direction. The edge thickness indicates the communication probability. The loops indicate autocrine circuits.
(B) Immunohistochemistry staining for SEMA4D (Left) and PLXNB2 (Right) in TH-*MYCN* bone.
(C) Quantification of percentage of positive cells stained for SEMA4D. The number of positive cells was determined using QuPath v0.5.1. Mean ± SD is presented in the graphs (Mann–Whitney U test, P = 0.0043).
(D) Kaplan-Meier curve showing the overall survival in the Kocak cohort (n= 649, GEO: GSE45547), separated by average *TGM2* expression. Yellow, *TGM2* above average (high); blue, *TGM2* below average (low).
(E) Kaplan-Meier curve showing the overall survival in the NRC cohort (n= 283, GEO: GSE85047), separated by average *GPR56/ADGRG1* expression. Magenta, *GPR56* above average (high); blue, *GPR56* below average (low).

**Supplementary Figure S13 | Alignment of bone marrow and matched TH-*MYCN* tumor microenvironment.**

(A) Joint alignment of TH-*MYCN* bone marrow and matched tumor microenvironment cells from five mice. Major cell populations are colored and labelled.
(B) UMAP embedding colored by sample fraction; bone marrow (BM) in magenta and tumors in blue,
(C) grouped by sample type; BM on the left and tumor on the right. Major cell populations are colored.
(D) Joint embedding representing the myeloid cluster by sample fraction (Left) and subpopulations (Right).
(E) Heatmap showing expression of top differentially expressed genes across the myeloid subpopulations (top color bar, as in UMAP clustering on Suppl. Fig. S5D)
(F) Feature plots of key marker gene expression for different myeloid subpopulations.
(G) Immunostaining for CD31/PECAM1 (magenta) and CD68 (green) in a TH-*MYCN* tumor. Arrows, CD31+CD68+ double-positive cell.

**Supplementary Figure S14 | Survival analysis for *NR2F6*, and *TREML4*** **expression.**

(A) Overall survival analysis of bulk RNA-seq data from the SEQC-498 cohort (GSE49711), stratified by *MYCN* amplification status. Kaplan–Meier curves show overall survival according to *NR2F6*, and *TREML4* median expression.
(B) Boxplots show *NR2F6*, and *TREML4* expression levels stratified by INSS stage.

**Supplementary Figure S15 | Myeloid signature in human neuroblastoma bone marrow metastasis.**

(A) UMAP embedding of 37,406 human bone marrow cells from 15 neuroblastoma patients from Mei et al. 2024. Top panel represents major cell populations, which are labelled and colored. Bottom panel represents neutrophil and myeloid cell subpopulations, shown on the combined UMAP embedding.
(B) Feature plot of *CD300E*, *PECAM1* and *SIGLEC10* expression, shown on the combined UMAP embedding.
(C) Heatmap of top 100 differentially expressed genes for the macrophage (Macro) subpopulation.
(D) UMAP embedding of subcluster view of the myeloid cells and neutrophils.
(E) Feature plot of *CD300E*, *PECAM1*, *SIGLEC10* and *C1QA* expression, as shown on a myeloid-specific joint embedding.
