# Supplementary material for: Immune remodeling and myeloid signature in the bone marrow niche of MYCN-driven neuroblastoma
Source: BJC Rep. 2026 Sep 15;4:46. doi: 10.1038/s44276-026-00249-3 (PMC13578270; doi:10.1038/s44276-026-00249-3)
Supplement: Supplementary file 4 [file 44276_2026_249_MOESM4_ESM.docx]

**Supplementary tables**

**Table S1.** Characteristics of TH-*MYCN* mouse samples used for scRNA-seq.

| Cell count | Sample ID | Genotype | Sex | Age (Days) |
| --- | --- | --- | --- | --- |
| 4327 | Het1_bm | Hemizygous | Male | 58 |
| 5460 | Het2_bm | Hemizygous | Female | 97 |
| 5255 | Homo1_bm | Homozygous | Female | 41 |
| 4170 | Homo2_bm | Homozygous | Male | 37 |
| 1702 | Homo3_bm | Homozygous | Female | 40 |
| 6067 | Wt1_bm | Wild-type | Female | 90 |
| 4263 | Wt2_bm | Wild-type | Female | 46 |
| 3155 | Wt3_bm | Wild-type | Female | 48 |

**Table S2.** Bone marrow cell type annotation.

| Gene | Annotation | Cluster number | Cell Count | Ref (PMID) |
| --- | --- | --- | --- | --- |
| *Tnfrsf13c* | Immature B cells | 0 | 4616 | [34824268](https://pubmed.ncbi.nlm.nih.gov/34824268) |
| *Arl5c* | Pre B cells | 1 | 4105 | [34824268](https://pubmed.ncbi.nlm.nih.gov/34824268) |
| *Cebpd* | Mature Neutrophils 2 | 2 | 3493 | [29466759](https://pubmed.ncbi.nlm.nih.gov/29466759/) |
| *Mmp8* | Mature Neutrophils 1 | 3 | 3468 | [29466759](https://pubmed.ncbi.nlm.nih.gov/29466759/) |
| *Ltf* | Immature Neutrophils | 4 | 2812 | [29466759](https://pubmed.ncbi.nlm.nih.gov/29466759/) |
| *Ltf* | Immature Neutrophils | 5 | 2217 | [29466759](https://pubmed.ncbi.nlm.nih.gov/29466759/) |
| *Arl5c* | Pre B cells | 6 | 2182 | [34824268](https://pubmed.ncbi.nlm.nih.gov/34824268) |
| *Prtn3* | Hematopoietic precursor cells | 7 | 1751 |  |
| *Mki67* | Cycling Pro B cells 3 | 8 | 1403 | [34824268](https://pubmed.ncbi.nlm.nih.gov/34824268) |
| *Cd68* | Macrophages | 9 | 1322 |  |
| *Fcnb* | Cycling Neutrophil Progenitors | 10 | 1301 | 32579887 |
| *Bank1* | Mature B cells | 11 | 1095 | [34824268](https://pubmed.ncbi.nlm.nih.gov/34824268) |
| *Cd3g* | T/NK cells | 12 | 783 |  |
| *Bok* | Cycling Pro B cells 1 | 13 | 749 | [34824268](https://pubmed.ncbi.nlm.nih.gov/34824268) |
| *E2f1* | Cycling Pro B cells 2 | 14 | 698 | [34824268](https://pubmed.ncbi.nlm.nih.gov/34824268) |
| *Vpreb1* | Pro B cells | 15 | 691 | [34824268](https://pubmed.ncbi.nlm.nih.gov/34824268) |
| *Siglech* | DC | 16 | 500 |  |
| *Cd300e* | Myeloid | 17 | 471 |  |
| *Ermap* | Proerythroblasts/Mast cells | 18 | 464 | [Bioarchive](https://www.biorxiv.org/content/10.1101/2021.08.12.456098v1.full) |
| *Arl5c* | Pre B cells | 19 | 141 | [34824268](https://pubmed.ncbi.nlm.nih.gov/34824268) |
| *Arl5c* | Pre B cells | 20 | 137 | [34824268](https://pubmed.ncbi.nlm.nih.gov/34824268) |
|  |  | Total | 34399 |  |

**Table S3.** TH-*MYCN* tumor cell type annotation.

| Gene | Annotation | Cluster number | Count |
| --- | --- | --- | --- |
| *Nefl* | G1 phase Sympathoblasts | 0 | 8080 |
| *Nefl* | G1 phase Sympathoblasts | 1 | 6968 |
| *Top2a* | S phase Sympathoblasts | 2 | 6330 |
| *Cdc20* | M phase Sympathoblasts | 3 | 5263 |
| *Chga* | Chromaffin-Sympathoblast | 4 | 4907 |
| *Ppia* | Sympathoblasts | 5 | 2402 |
| *Igkc* | B cells | 6 | 2071 |
| *Cd3d* | T cells | 7 | 1404 |
| *Fcer1g* | Myeloid | 8 | 1327 |
| *Aspm* | G2M phase Sympathoblasts | 9 | 844 |
| *Ptprs* | Transitioning | 10 | 627 |
| *S100a9* | Neutrophils | 11 | 425 |
| *Cdh5* | Endothelial | 12 | 367 |
| *Siglech* | pDC | 13 | 242 |
| *Nefl* | G1 phase Sympathoblasts | 14 | 233 |
| *Syngr2* | mDC | 15 | 210 |
| *Mgp* | Mesenchymal | 16 | 201 |
| *Th* | Chromaffin | 17 | 192 |
| *Fcer1g* | Myeloid | 18 | 135 |
| *Cd3d* | T cells | 19 | 104 |
|  |  | Total | 42332 |

**Table S4.** Heatmap list of genes

| Gene | Annotation |
| --- | --- |
| *MYCN* | G1 phase Sympathoblasts |
| *Phox2b* | G1 phase Sympathoblasts |
| *Isl1* | G1 phase Sympathoblasts |
| *Stmn1* | G1 phase Sympathoblasts |
| *Nefm* | G1 phase Sympathoblasts |
| *Nefl* | G1 phase Sympathoblasts |
| *Cenpa* | M phase Sympathoblasts |
| *Ube2c* | M phase Sympathoblasts |
| *Cdc20* | M phase Sympathoblasts |
| *Rrm2* | S phase Sympathoblasts |
| *Hist1h1b* | S phase Sympathoblasts |
| *Top2a* | S phase Sympathoblasts |
| *Cdk1* | S phase Sympathoblasts |
| *Prc1* | S phase Sympathoblasts |
| *Atad2* | S phase Sympathoblasts |
| *Esco2* | S phase Sympathoblasts |
| *Aspm* | G2M phase Sympathoblasts |
| *Cenpe* | G2M phase Sympathoblasts |
| *Arl6ip1* | G2M phase Sympathoblasts |
| *Mis18bp1* | G2M phase Sympathoblasts |
| *Nusap1* | G2M phase Sympathoblasts |
| *Kif20b* | G2M phase Sympathoblasts |
| *Hmmr* | G2M phase Sympathoblasts |
| *Ckap2l* | G2M phase Sympathoblasts |
| *Cenpf* | G2M phase Sympathoblasts |
| *Kif23* | G2M phase Sympathoblasts |
| *Pimreg* | G2M phase Sympathoblasts |
| *Sgo2a* | G2M phase Sympathoblasts |
| *Chga* | Chromaffin-Sympathoblast |
| *Th* | Chromaffin |
| *Caly* | Chromaffin |
| *Dbh* | Chromaffin |
| *Cdkn1c* | Chromaffin |
| *Ptprs* | Transitioning |
| *Gm26917* | Transitioning |
| *Zdbf2* | Transitioning |
| *Eml5* | Transitioning |
| *Dst* | Transitioning |
| *Nktr* | Transitioning |
| *Xist* | Transitioning |
| *Zbtb20* | Transitioning |
| *Sox11* | Transitioning |
| *Nav1* | Transitioning |
| *Tcf4* | Transitioning |
| *Mgp* | Mesenchymal |
| *Prrx1* | Mesenchymal |
| *Pdgfrb* | Mesenchymal |
| *Dcn* | Mesenchymal |
| *Col1a1* | Mesenchymal |
| *Col1a2* | Mesenchymal |
| *Col14a1* | Mesenchymal |
| *Egfl7* | Endothelial |
| *Cdh5* | Endothelial |
| *Gpihbp1* | Endothelial |
| *Plvap* | Endothelial |
| *Emcn* | Endothelial |
| *Cd79a* | B cells |
| *Igkc* | B cells |
| *Ebf1* | B cells |
| *Ighm* | B cells |
| *Cd79b* | B cells |
| *Ly6d* | B cells |
| *Ms4a1* | B cells |
| *Iglc2* | B cells |
| *Ighd* | B cells |
| *Mzb1* | B cells |
| *Bank1* | B cells |
| *Ms4a4b* | T cells |
| *Trbc2* | T cells |
| *Cd3d* | T cells |
| *Cd3g* | T cells |
| *Trac* | T cells |
| *Skap1* | T cells |
| *Lat* | T cells |
| *Cd28* | T cells |
| *Lef1* | T cells |
| *Lck* | T cells |
| *Trbc1* | T cells |
| *Tcf7* | T cells |
| *AW112010* | T cells |
| *Cd3e* | T cells |
| *Itk* | T cells |
| *Cd68* | Myeloid |
| *Lyz2* | Myeloid |
| *Fcer1g* | Myeloid |
| *Msrb1* | Myeloid |
| *Pla2g7* | Myeloid |
| *Lst1* | Myeloid |
| *Clec4a3* | Myeloid |
| *Csf1r* | Myeloid |
| *Cd300c2* | Myeloid |
| *Fcgr4* | Myeloid |
| *Clec4a1* | Myeloid |
| *Ms4a6d* | Myeloid |
| *Igsf6* | Myeloid |
| *S100a9* | Neutrophils |
| *S100a8* | Neutrophils |
| *Mmp9* | Neutrophils |
| *Cxcr2* | Neutrophils |
| *Syngr2* | mDC |
| *Ahr* | mDC |
| *Clec10a* | pDC |
| *Siglech* | pDC |

**Table S5.** Primary antibodies for IHC and IF.

| Target | Concentration or Dilution | Product number | Supplier | Application |
| --- | --- | --- | --- | --- |
| CEACAM1 | 1:100 dilution | HPA011041 | Human protein atlas/Merck/Sigma-Aldrich | IHC |
| DC-SIGN (CD209) | 1:100 dilution | MA5-35828 | Invitrogen | IHC |
| SEMA4D | 1:100 dilution | ab134128 | abcam | IHC |
| PLXNB2 | 3 µg/mL | AF5329 | R&D Biotechne | IHC |
| TGM2 | 0.2 µg/mL | AF4376 | R&D Biotechne | IF |
| GPR56 (ADGRG1) | 2 µg/mL | 720286 | Invitrogen | IF |
| FABP4 | 10 µg/mL | AF1443 | R&D Biotechne | IF |
| CD68 | 1:400 dilution | 97778 | CST | IF |
| CD31 (PECAM1) | 10 µg/mL | AF3628 | R&D Biotechne | IF |

**Table S6.** Secondary antibodies for IF.

| Antibody | Dilution | Supplier | Catalog # |
| --- | --- | --- | --- |
| donkey anti-rabbit 488 | 1:1000 | Invitrogen | A21206 |
| donkey anti-sheep 555 | 1:1000 | Invitrogen | A21436 |
| donkey anti-goat 555 | 1:1000 | Invitrogen | A32816 |
